# Impact of Subspecialty Pediatric Palliative Care on Children with Heart Disease; A Systematic Review and Meta-analysis

**DOI:** 10.1007/s00246-024-03535-4

**Published:** 2024-06-22

**Authors:** James Ting, Kathryn Songer, Valerie Bailey, Chloe Rotman, Stuart Lipsitz, Abby R. Rosenberg, Claudia Delgado-Corcoran, Katie M. Moynihan

**Affiliations:** 1 Department of Pediatrics, Johns Hopkins University, Baltimore, MD USA; 2 Department of Pediatrics, University of Utah, Salt Lake City, UT USA; 3 Department of Cardiology, Boston Children’s Hospital, MS BCH3215, 300 Longwood Ave, Boston, MA 02115 USA; 4 Medical Library, Boston Children’s Hospital, Boston, MA USA; 5 Department of General Internal Medicine and Primary Care, Center for Patient Safety, Research, and Practice, Brigham and Women’s Hospital, Boston, MA USA; 6 Department of Pediatrics, Harvard Medical School, Boston, MA USA; 7 Department of Psychosocial Oncology and Palliative Care, Dana-Farber Cancer Institute, Boston, MA USA; 8 Department of Pediatrics, Boston Children’s Hospital, Boston, MA USA; 9 Children’s Hospital at Westmead Clinical School, Faculty of Medicine and Health, The University of Sydney, Sydney, NSW Australia

**Keywords:** Child, Palliative care, Outcome assessment, Health care, Heart diseases

## Abstract

While many experts in pediatric cardiology have emphasized the importance of palliative care involvement, very few studies have assessed the influence of specialty pediatric palliative care (SPPC) involvement for children with heart disease. We conducted a systematic review using keywords related to palliative care, quality of life and care-satisfaction, and heart disease. We searched PubMed, EMBASE, CINAHL, CENTRAL and Web of Science in December 2023. Screening, data extraction and methodology followed Preferred Reporting Items for Systematic Reviews and Meta-Analyses (PRISMA) recommendations. Pairs of trained reviewers independently evaluated each article. All full texts excluded from the review were hand-screened for eligible references including systematic reviews in general pediatric populations. Two reviewers independently extracted: (1) study design; (2) methodology; (2) setting; (3) population; (4) intervention/exposure and control definition; (5) outcome measures; and (6) results. Of 4059 studies screened, 9 met inclusion criteria including two with overlapping patient data. Study designs were heterogenous, including only one randomized control and two historical control trials with SPPC as a prospective intervention. Overall, there was moderate to high risk of bias. Seven were single centers studies. In combined estimates, patients who received SPPC were more likely to have advance care planning documented (RR 2.7, [95%CI 1.6, 4.7], *p* < 0.001) and resuscitation limits (RR 4.0, [2.0, 8.1], *p* < 0.001), while half as likely to have active resuscitation at end-of-life ([0.3, 0.9], *p* = 0.032). For parental stress, receipt of SPPC improved scores by almost half a standard deviation (RR 0.48, 95%CI 0.10, 0.86) more than controls. Ultimately, we identified a paucity of high-quality data studying the influence of SPPC; however, findings correlate with literature in other pediatric populations. Findings suggest benefits of SPPC integration for patients with heart disease and their families.

**Supplementary Information:**

The online version contains supplementary material available at 10.1007/s00246-024-03535-4.

## Introduction

Advancements in pediatric cardiology continue to improve outcomes for children with advanced heart disease (AHD) and their families [1–3]. These children are living longer, frequently with more medical complexity, and their families may benefit from support from palliative care [4, 5]. Palliative care provides an interdisciplinary approach to mitigate pain and suffering and to improve the emotional wellbeing and quality of life of seriously ill patients and their caregivers [6–9]. Many organizations and leaders within pediatric cardiology have acknowledged palliative care as an important aspect of care for children with AHD [5, 10, 11]. Specific needs have been described for cardiac conditions with high mortality rates [e.g., single ventricle heart disease (SVHD) and end-stage heart failure including patients being bridged with ventricular assist device (VAD)] [5, 11–14]. However overall referral rates for palliative care in pediatric heart disease lag far behind other conditions [10].

While palliative care for children with AHD can be delivered by several models [15, 16], research focuses on consultative subspecialty pediatric palliative care (SPPC) models delivered by a specialized team of interdisciplinary clinicians who provide a range of interventions/services encompassing but not limited to psychosocial support, symptom management, care coordination, hospice services and bereavement [17]. Prior systematic reviews have studied SPPC delivery in a broad range of populations, including oncology, chronic diseases, and neonatology, finding improved quality of life, improved caregiver experience, and higher rates of advance care planning (ACP) [18–20].

Limited empiric data characterize impacts of SPPC in pediatric heart disease, despite it being widely identified as a key area of further research [5, 10–14, 21–25]. We sought to describe the published literature assessing the influence of SPPC on the various aspects of care for children with AHD such as advanced care planning and location of death.

## Methods

We utilized the Preferred Reporting Items for Systematic Reviews and Meta-Analyses (PRISMA) recommendations (Supplementary Table 2), conducted this review in accordance with the Declaration of Helsinki and registered the study on PROSPERO (https://www.crd.york.ac.uk/prospero/, CRD42023484694).

### Selection Criteria

We included studies meeting the following criteria: (1) study design: randomized controlled trials (RCT) and comparative observational studies, including cohort, case–control, before-after studies, and cross-sectional studies. Case series or non-comparative cross-sectional studies were excluded; (2) population: pediatric patients age < 21 with a cardiovascular disease diagnosis. Studies that did not limit to this population were included if results were stratified by diagnostic category; (3) intervention/exposure: presence of SPPC as a broad concept encompassing specialized services provided as part of a ‘palliative care team/program’; (4) control: usual/standard care or no SPPC; (5) setting: inpatient, intensive care, ambulatory, community-based, home, or hospice; (6) outcomes: any patient-level (e.g., survival, and mode of death), family-level (e.g., stress, depression), staff-level (e.g., perceptions of end-of-life experience), or health-system-level outcome (e.g., costs); and (7) publication status and language: peer-reviewed studies published in English or Spanish.

### Search Strategy

The search was conducted in December 2023 by a medical librarian, using PubMed, EMBASE, CINAHL, Web of Science and Cochrane Central Register of Controlled Trials (CENTRAL). The search strategy included extensive keywords related to palliative care, quality of life and care-satisfaction, and heart disease; terms included, but were not limited to, “palliative care,” “hospice care,” “terminal care,” “quality of life,” “personal satisfaction,” “mental stress,” “congenital heart defect,” and “heart disease” (Supplementary Table 3). Publication date was limited from 2000 through the search date. In EMBASE, the search was limited to exclude conference abstracts. Results were exported to, and deduplicated in EndNote [26].

### Study Screening and Selection

All articles were uploaded to Covidence (https://www.covidence.org, Veritas Health Innovation, Melbourne, Australia) for screening and data extraction. Pairs of trained reviewers (JT, KS, VB, CDC, KM) independently evaluated each article generated by the search strategy by the title and abstract. Discrepancies between reviewers were discussed until a consensus was reached among study authors. All full texts excluded from the review were hand-screened for potentially eligible references including systematic reviews in general pediatric populations [19].

### Data Extraction

For each article, we collected: (1) study design; (2) methodology; (2) setting; (3) study population; (4) definitions of intervention/exposure and control; (5) outcome measures; and (6) results. Two reviewers independently extracted data (JT, KS, VB, CDC), with conflicts resolved by KM. Study authors were contacted for additional data unavailable in the published articles.

### Risk of Bias Assessment

Risk of bias was assessed including reported conflicts of interest and the Quality in Prognosis Studies or the revised Cochrane risk of bias for RCT tools, as appropriate [27, 27–29]. Domains were: (1) study participation, (2) study attrition, (3) prognostic factor measurement, (4) outcome measurement, (5) confounder measurement, (6) statistical analysis and reporting domains. A composite quality score was calculated by assigning 0–2 points for high, moderate and low risk of bias in each category (maximum of 12 as low bias, high quality).

### Statistical Analysis and Meta-analysis

Total SPPC referral rates were calculated with numerator, denominator and exact binomial confidence intervals overall and restricted to decedents, excluding the prospective interventional studies.

We performed a meta-analysis to test for homogeneity of effects across studies and obtain a pooled estimate of treatment effect over the individual studies. Studies with duplicate/overlapping patient data were excluded from meta-analyses. For dichotomous outcomes, we estimated the relative risk for intervention vs control; for time-to-event outcomes (surgical and length of stay), we estimated the ratio of means or medians for intervention vs control (under an accelerated time-to-event model the ratio of means and medians are the same) [30].

For studies with continuous outcome measures that used different scoring systems we used Cohen’s *d* as the intervention effect in each study. Cohens *d* is the difference in means in intervention versus control divided by the pooled standard deviation, and thus can be interpreted as the differences in intervention versus control in terms of standard deviations with appropriate effect size cutoffs [31]. This measures an intervention effect in a study that is not dependent on the scoring system/measure itself, and thus can be compared/combined across studies. These studies with continuous outcomes were pre/post studies, so the intervention effect is Cohen’s *d* in terms of difference-in-differences (mean ‘pre minus post’ difference in intervention, minus mean ‘pre minus post’ difference in control). The DerSimonian and Laird test was used to test for homogeneity of intervention effect across studies [32]. If the ‘homogeneity test’ was rejected combined risk ratio probabilities were not presented. If homogeneity was established; the pooled estimate was determined as a weighted average of the intervention effect estimates from the individual studies [33]. The weights used in the pooled estimate equal the inverse of the variance of the estimated intervention effect from each study. Given the small number of studies, the meta-analysis was performed assuming a fixed effect for each study. We determined the 95% confidence interval for the pooled intervention effects, as well as a *p* value for the null hypothesis that the pooled intervention effect equals the null value. A forest plot was used to display the intervention effect for each individual study and the pooled estimate.

## Results

### Study Characteristics

**Fig. 1:**
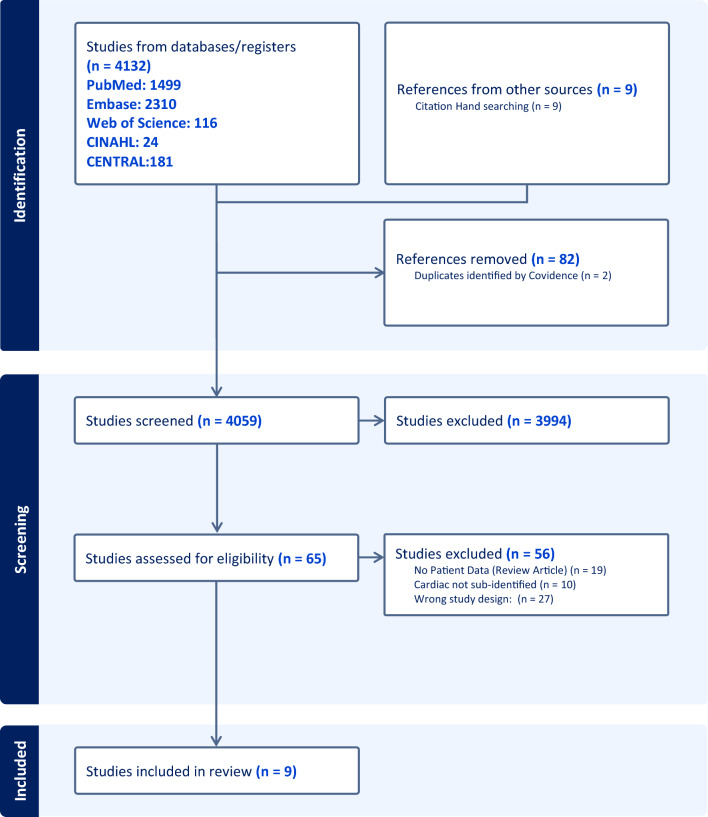
PRISMA flowchart for study selection. PRISMA flowsheet showing initial inclusion of 4059 articles, 65 assessed for eligibility, and 9 studies ultimately included for review

Of 4059 studies screened and 65 assessed for eligibility at full-text, nine met study inclusion criteria including two with overlapping patient data (Fig. 1) [34–42]. All studies were from the United States. Six were retrospective cohort observational studies with chart review to determine SPPC involvement as part of clinical care, including two with additional cross-sectional survey components (one of staff and one of bereaved parents) [35–39, 41]. Three involved SPPC consultation as a prospective intervention (two historical control trials and one randomized control trial) [34, 40, 42]. Studying outcomes in cardiac disease according to SPPC involvement was the primary aim in six (66.7%) [35–37, 40–42]. Seven (77.8%) were single center studies [35–37, 39–42]. Only one included all disease groups and sub-defined results for cardiac patients [34]. Of the remaining, five included all cardiac diseases [35–39] (three focused on cardiac intensive care unit [CICU] patients) [35, 36, 39], one was in VAD patients [41] and two were in neonates [40, 42] (one restricted to SVHD) [40]. Population sample sizes are shown (Table 1). Overall, most studies had moderate- to high-risk for bias (Table 1). While the prospective interventional study populations were well-matched, no study performed multivariable analyses [34, 40, 42].

**Table 1:** Characteristics of included studies

Author/year	Study design	Study years	Centers^a^	Survivors/decedents	Population description	# of Cardiac patients	# (%) No SPPC	# (%) with SPPC	Quality score
Delgado-Corcoran 2021^bc^	Retrospective observational cohort study	2014–2017	Single, inpatient	Survivors and decedents	CICU patients < 21 yrs meeting CAPC criteria: CICU LOS > 14 days or ‚ > 3 hospitalizations in 6 months. Some analyses in decedents	288 (36 decedents)	240 (83%)	48 (17%)	4 High Bias/Low Quality
Moynihan 2021^b^	Retrospective observational cohort study	2007–2009 and 2015–2018	Single, inpatient	Decedents out-of-institution deaths in some analyses	Major cardiac disease necessitating hospitalization. SPPC analysis focuses on CICU decedents. < 21 years. Excluding deaths unrelated to cardiac disease	7441 inpatients (209 deaths with 186 in the CICU)	Overall 7250/7741 Decedents 133/209 (CICU 125/186)	Overall 191/7741 Decedents 76/209 (CICU 61/186)	5 Moderate Bias/Quality
Bailey 2022	Cross-sectional survey and Retrospective cohort study	2019–2021	Single, inpatient	Decedents	Restricted to patients with cardiac disease in the CICU with > 24 h CICU admission prior to death	60	28 (47%) Patients 290 (46%) Responses	32 (53%) Patients 347 (54%) Responses	5 Moderate Bias/Quality
Delgado-Corcoran 2020^bc^	Retrospective observational cohort study	2014–2017	Single, inpatient	Survivors and decedents	Restricted to patients in the CICU. < 21 years. Some outcomes were restricted to decedents	1389	1277 (92%) 43/85 Decedents	112 (8%) 42/85 Decedents	5 Moderate Bias /Quality
Hancock 2018^b^	Randomized Controlled Trial. Intervention was early SPPC input	2013–2015	Single, inpatient	Survivors and decedents	Patients with SVHD planned to undergo palliative surgery, prenatal to 30 days or hospital discharge and disease specific. Mothers only	38	20 (53%) (standard care) with 17 (50%) intervention	18 (47%) (early SPPC) with 17(50%) intervention	7 Moderate Bias/Quality
Moynihan 2022	Cross-sectional survey and Retrospective cohort study	2007–2010	Multi—2 centers, inpatient	Decedents—death 9 months to 4 years prior	Restricted to patients with cardiac disease. Any acquired or congenital heart disease. < 21 years. English speaking families only	44 complete responses of 128 (response rate 34.4%)	37 (84%)	7 (16%)	3 High Bias/Low Quality
Knoll 2020^b^	Retrospective observational cohort study	2014–2017	Single, inpatient	Survivors and decedents	Restricted to patients with cardiac disease on a VAD. < 21 years	55 (13 decedents)	32 (58%)	23 (42%)	4 High Bias/Low Quality
Callahan 2019^b^	Prospective Interventional Study with early Palliative care as the Baby, Attachment, Comfort Interventions	2017–2018 A Pre- and Study period (1 month washout)	Single, inpatient	Survivors—survey data was excluded for parents whose neonate died	Mothers and fathers of neonates with CHD in the NICU. Transferred neonates, limited English proficiency, or whose neonate was diagnosed with a life-limiting condition were excluded also those parents who declined participation due to psychological distress	77 parents and 53 infants	Parents: 51 (66%) Infants: 35 (66%)	Parents: 26 (34%) Infants: 18 (34%)	5 Moderate Bias/Quality
Gans 2016	Historical control trial. Enrollment in palliative care services	2010–2012	Multi: 11 California counties, home-based	Survivors and decedents	Children < 21 years, patients with cardiac disease defined. Patients had to have full-scope, no share-of-cost Medicaid, and an eligible “life limiting condition”, Included if enrolled > 60 days	12 (9% of total cohort)	Not defined	Not defined	3 High Bias/Low Quality

*SPPC* subspecialty pediatric palliative care, *CICU* cardiac intensive care unit, *CAPC* Center to Advance Palliative Care, *LOS* length of stay, *CHD* congenital heart disease, *SVHD* single ventricle heart disease, *VAD* ventricular assist device, *NICU* neonatal intensive care unit

^a^All studies were conducted in the United States

^b^Studying outcomes in cardiac disease according to SPPC involvement was the primary aim

^c^These studies had overlapping patient populations, only one was included in risk ratio outcome meta-analyses

### SPPC Referral Rates

Overall SPPC referral rate was 3.7% [95%CI 3.3, 4.1] (*n* = 326/8885), compared to 40.1% when restricted to decedents [95%CI 35.4, 40.1] (*n* = 165/411). Among studies of decedents, this ranged from 15.9% in the oldest study to 61.5% in the VAD study.

### Outcomes by SPPC

Clinical outcomes examined according to SPPC involvement are summarized in Table 2. Synthesis by clinical outcome is shown for findings restricted to decedents (Table 3) and overall (Table 4).

**Table 2:** Summary of outcomes by papers

Study ID	Study aim	Outcomes examined	Summary of key findings related to SPPC
Delgado-Corcoran 2021	Apply criteria from Center to Advance Palliative Care to a cohort of children treated in a CICU and compare children who received a SPPC to those eligible who did not	Mode of death, Location of death, Survival, Treatment of cardiac disease, Hospital and CICU LOS and Total hospitalizations	SPPC associated with higher disease burden (CICU and total hospital days, mortality) and less surgical interventions. Among decedents, SPPC was associated with higher hospital LOS, and no differences in location of death, or mode of death
Moynihan 2021	To characterize EOL care for children with AHD who die including intensity of interventions, mode of death, and costs, evaluating the influence of SPPC	Mode and location of death, LOS, hospital charges, advance care plans (ACP), resuscitation status, interventions prior to death	Most inpatient pediatric AHD deaths occurred in the CICU with a high disease burden at EOL. SPPC led to greater ACP and resuscitation documentation, less invasive therapies at EOL, and lower hospital costs
Bailey 2022	To evaluate Pediatric Intensive Care Unit Quality of Dying and Death (PICU-QODD) between disciplines and end-of-life circumstances	PICU-QODD score, quality of the moment of death, quality of the 7 days prior to death	SPPC prior to death was not significantly associated with differences in clinician PICU-QODD scores, or rating of the quality of the moment of death. However quality of life for the 7 days prior to death was rated higher with SPPC
Delgado-Corcoran 2020	Describe SPPC in children with heart disease; determine the impact of SPPC on end-of-life	Mode and Location of death, Survival, Treatment of cardiac disease, Hospital and CICU LOS and Total hospitalizations	SPPC was associated with greater LOS, higher mortality, and fewer surgical interventions. Among decedents, SPPC was associated with higher hospital LOS, higher rates of hospice, higher rates of comfort care or discontinuation of ICU therapies, and fewer deaths in ICU or with resuscitation
Hancock 2018	To examine the benefit of early pediatric palliative care consultation on maternal stress in mothers of infants with SVHD	Beck Depression Inventory-II, State-Trait Anxiety Index, Brief Cope Inventory, Parental health-related quality of life/family functioning, survival, LOS	SPPC was associated with less maternal anxiety, higher brief cope inventory positive reframing scores, and positive change in family impact module communication and family relationship scores
Moynihan 2022	To explore bereaved parents’ perceptions of a good death for children with heart disease	Parents agreement or disagreement that their child experienced a Good Death	SPPC involvement did not influence good death responses
Knoll 2020	To characterize use of SPPC services in children with VAD and impacts on end-of-life directives and clinical outcomes	Mode of death, advance care planning, resuscitation status, survival	Patients and families receiving SPPC services were more likely to limit resuscitation efforts and experience compassionate VAD withdrawal at the time of death
Callahan 2019	To employ early palliative care methods (called Baby, Attachment, Comfort Interventions) and assess psychological distress in parents of neonates with CHD	Parental stress (Neonatal Unit Parental Stressor Scale NUPS), Depression Anxiety Stress Index-21 (DASS-21), LOS	SPPC reduced parental stress, especially in measures of perception of the neonate’s pain and discomfort, the experience of being separated from the neonate, and the experience of being unsupported. However, depression or anxiety did not change
Gans 2016	To assess a change from before SPPC enrollment in: 1) costs per enrollee per month, 2) costs by service type (inpatient, outpatient, emergency department, and pharmacy) and diagnosis category, and 3) utilization (inpatient care days)	Cost Distribution, Mean Inpatient Days	The PFC model (Partners for Children; provision of home-based therapeutic services, 24/7 access to medical advice, and personally tailored care coordination) reduces costs for children with life-limiting conditions

*SPPC* subspecialty pediatric palliative care, *CICU* cardiac intensive care unit, *LOS* length of stay, *EOL* end-of-life, *AHD* advanced heart disease, *ICU* intensive care unit, *SVHD* single ventricle heart disease, *VAD* ventricular assist device, *CHD* congenital heart disease

**Table 3:** Summary of decedent outcomes

Level	Outcome	Study	Results. *N* (%) or Median (IQR) unless stated
Patient	Mode of death	Delgado-Corcoran 2021	Active resuscitation 2 (10%) SPPC vs 5 (31%) no SPPC, withdrawal of LST 14 (70%) SPPC vs 10 (63%) No SPPC, comfort care 4 (20%) SPPC vs 0% No SPPC, unknown 0% vs 1 (6%) no SPPC. SPPC had no statistical difference with mode of death (*p* = 0.078)
		Moynihan 2021	Active resuscitation in 5 (8%) SPPC vs 18 (14%) no SPPC, vs discontinuation of LST 41 (67%) SPPC, 90 (72%) No SPPC, Non-escalation 15 (25%) SPPC 15 (12%), Brain death 2 (2%) no SPPC. SPPC had no statistical association with mode of death (*p* = 0.1)
		Delgado-Corcoran 2020	Active resuscitation in 5 (12%) SPPC vs 11 (26%) no SPPC, withdrawal of LST 21 (50%) SPPC, 28 (65%) no SPPC, Comfort care 15 (36%) SPPC 1 (2%) no SPPC. SPPC associated with a difference in mode of death (*p* = 0.002)
		Knoll 2020	Full resuscitation/continued care at EOL in 2 (25%) SPPC, 5 (100%) no SPPC vs compassionate discontinuation/hospice: 6 (75%) SPPC and 0 SPPC. Patients receiving SPPC services were more likely to die with goals-of-care being palliative vs curative (*p* = 0.04)
	Location of death	Delgado-Corcoran 2021	ICU 15 (75%) SPPC vs 12 (75%) No SPPC, Home with hospice care 3 (15%) SPPC vs 0%, hospital or LTAC 2 (10%) vs 3 (19%), unknown 0 vs 1 (6%) no SPPC. SPPC associated with no statistical difference in location of death (*p* = 0.286)
		Moynihan 2021	CICU 61 (69%) SPPC 125 (82%), Hospital 28 (31%) SPPC, 28 (18%) no SPPC, Institutional hospice 3 (3%) SPPC vs 0. (*p* value not reported)
		Delgado-Corcoran 2020	ICU 28 (67%) SPPC vs 31 (72%) no SPPC, home with hospice care 10 (24%) SPPC vs 1 (2%) no SPPC, hospital 2 (5%) SPPC vs 8 (19%) no SPPC, home unexpected 1 (2%) SPPC vs 2 (5%) no SPPC, chronic care facility 1 (2%) SPPC vs 0%, unknown 0% vs 1 (2%) no SPPC. SPPC associated with a difference in location of death (*p* = 0.020)
	Advance Care Plan (ACP)	Moynihan 2021	CICU decedents: 40 (66%) SPPC vs 38 (30%) no SPPC. Out of hospital deaths ACP in 9 (50%) SPPC, 0% no SPPC. SPPC was associated with greater the ACP meeting frequency (RR 2.2 [95% CI 1.6, 3.0]) (*p* < 0.001)
		Knoll 2020	ACP in 3 (13%) with SPPC vs 1 (3%) no SPPC. (*p* = NS value not reported)
	Resuscitation status	Moynihan 2021	Documentation of DNR/DNI Resuscitation status in 35 (57%) SPPC vs 20 (16%) no SPPC. SPPC was associated with higher DNR/DNI status RR 3.6 [95% CI 2.3–5.7]) (*p* < 0.001)
		Knoll 2020	Documented DNR/DNI resuscitation status in 6 (26%) with SPPC vs 1 (3%) no SPPC. A statistically significant increase in DNR/DNI status with SPPC (*p* = 0.017)
	Length of stay -Hospital days	Moynihan 2021	Decedents: 78.3 (33.225 172.55) SPPC vs 14.2 (0.95 55.8) no SPPC
		Delgado-Corcoran 2020	Decedents: 58 (19–90) SPPC vs 18 (3–35) no SPPC. Statistically higher hospital LOS for decedents (*p* = 0.001)
		Delgado-Corcoran 2021	Decedents 85 (58–118) SPPC vs 46 (22–92). Statistically higher hospital LOS for decedents (*p* = 0.027)
	Length of stay—CICU days	Delgado-Corcoran 2021	Decedents 43 (21–56) vs 23 (17–45). SPPC not associated with greater CICU LOS (*p* = 0.340)
		Delgado-Corcoran 2020	Decedents 16 (5–52) SPPC vs 10 (3–22) No SPPC. In decedents CICU LOS was NS (*p* = 0.055)
Parent	Good Death perceptions	Moynihan 2022	6 (86%) with SPPC agreed (5 strongly and 1 somewhat) vs 25 (68%) No SPPC (11 strongly, 14 somewhat). 1 (14%) with SPPC disagreed (somewhat) vs 12 (32%) No SPPC (6 strongly, 6 somewhat). SPPC had no statistical association with good death response (*p* = 0.3)
Staff	PICU QODD	Bailey 2022	Of 713 surveys Mean (SD) PICU-QODD score was 89.7 (9.2) with SPPC vs 89.5 (10.2) without. No statistical difference in mean PICU-QODD scores (mean difference 0.1 (− 2.6 to 2.8)
	Quality of 7 days prior	Bailey 2022	Quality of life for the 7 days prior to death was rated 5 [3–7] SPPC, versus 4 [2–6] no SPPC. Quality of life for the 7 days prior to death was rated lower for No SPPC (p = 0.02)
	Moment of death	Bailey 2022	Moment of death was rated 9 with SPPC [6, 10] and 9 No SPPC [8, 10]. Moment of death quality rating was similar (*p* = 0.42)
System	Hospital charges (day of death, 7 days prior, admission)	Moynihan 2021	Median hospital charges on the day of death $8243 [IQR $2632‚$19 261] versus median $13,301 [IQR$7342‚$23 488] and 7 days before (median $151 357 [IQR $105 073‚ $211 905] versus median $125 723 [IQR $73 737‚ $176 703]. In patients without SPPC involvement, median hospital charges were 61% higher on the day of death (*p* = 0.016) and 20% higher in the 7 days before (*p *= 0.021), whereas total admission costs were comparable

*IQR* interquartile range, *SPPC* subspecialty pediatric palliative care, *LST* life sustaining treatment, *EOL* end of life, *LTAC* long-term acute care, *ICU* intensive care unit, *ACP* advance care planning, *DNR* do not resuscitation, *DNI* do not intubate, *LOS* length of stay, *CICU* cardiac intensive care unit, *NS* non significant

**Table 4:** Summary of combined (survivor and decedent) outcomes

Level	Outcome	Study	Results. *N* (%) or Median (IQR) unless stated
Patient	Length of stay-Hospital days	Gans 2016	Mean number of days inpatient pre- and post-enrollment was 7.6 and 1.5, respectively
		Delgado-Corcoran 2020	Overall: 60 days (22–100) SPPC vs 7 (3–19) No SPPC. Statistically higher hospital LOS overall (*p* < 0.001)
		Hancock 2018	SPPC 25 (19–31) vs 32 (23–79). No statistical difference in LOS (*p* = 0.13)
		Callahan 2019	Mean (SD) 24 (22) SPPC, vs 33 (46) no SPPC. No statistical difference in LOS (*p* = 0.37)
		Delgado-Corcoran 2021	Overall: 91 (58–150) SPPC, 35 (24–55) No SPPC. Statistically higher hospital LOS overall (*p* < 0.001)
	Length of stay—CICU days	Delgado-Corcoran 2021	Overall: 27 (14–54) SPPC vs 17 (10–25) No SPPC. SPPC associated with greater CICU LOS overall (*p* < 0.001)
		Hancock 2018	15 (12–18) SPPC vs 20 (12–43) no SPPC. No statistical difference in LOS (*p* = 0.19)
		Delgado-Corcoran 2020	Overall: 11 (5–30) SPPC vs 2 (1–7) No SPPC with statistically higher total CICU days (*p* < 0.001)
	Survival	Delgado-Corcoran 2021	Mortality 20 (42%) SPPC vs 16 (7%) no SPPC. SPPC associated with higher unadjusted mortality (*p* < 0.001)
		Delgado-Corcoran 2020	Mortality 42 (38%) SPPC vs 43 (3%) no SPPC. SPPC associated with higher unadjusted mortality (*p* < 0.001)
		Knoll 2020	Mortality 8 (33%) SPPC vs 5 (15%) no SPPC. No statistical difference (*p* value not reported)
		Hancock 2018	Mortality 2 (11%) SPPC vs 4 (20%) no SPPC. No statistical difference in mortality (*p* = 0.66)
	Treatment of cardiac disease	Delgado-Corcoran 2021	Surgical Intervention (vs medical/catheter-based) 39 (81%) SPPC vs 231 (96%) no SPPC. SPPC associated with non-surgical treatment approaches to heart disease (*p* = 0.001)
		Delgado-Corcoran 2020	Surgical Intervention (vs medical/catheter-based) 75 (67%) SPPC vs 992 (78%) no SPPC. SPPC associated with non-surgical treatment approaches to heart disease (*p* = 0.012)
	Resuscitation limitations	Knoll 2020	Full code in 17 (74%) SPPC vs 31 (97%) no SPPC. No statistical difference (*p* value not reported)
Survey-Parent Mean (SD)	Parental stress	Hancock 2018	State-Trait Anxiety Index1 State: pre/post SPPC Intervention scores 48.2 (11.7) down to 40.6 (9.5) No SPPC 45.2 (14.0) down to 45.5 (14.0). A significant reduction in maternal anxiety in the early SPPC group scores reduced by 7.6 points versus 0.3 points with no SPPC (*p* = 0.02)
		Callahan 2019	Neonatal Unit Parental Stressor Scale pre/post SPPC Intervention scores: 3.58 (1.21) to 2.92 (− 1.20) vs No SPPC 3.28 (1.39) to 3.16 (1.15). Parental stress decreased pre/post with SPPC (*p* = 0.01, Effect size 0.53) but not in No SPPC (*p* = 0.40)
		Callahan 2019	Depression Anxiety Stress Index-21 Stress pre/post SPPC Intervention scores: 4.6 (3.89) to 3.48 (3.06). Stress 4.73 (4.0) to 4.39 (4.0). Stress decreased pre/post with SPPC (*p* < 0.01) but not in the control group (*p* = 0.47). Effect size 0.30
	Depression	Hancock 2018	Beck Depression Inventory-II pre/post SPPC Intervention scores 10.4 (6.4) and no SPPC score 10.3 (8.5). No significant difference. *p* value = 0.98
		Callahan 2019	Depression Anxiety Stress Index-21 pre/post SPPC Intervention scores 1.88 (1.88) to 1.76 (2.73). No SPPC Depression 1.78 (2.18) to 1.75 (2.76). No significant difference (*p* value not reported)
	Anxiety	Callahan 2019	Depression Anxiety Stress Index-21 Anxiety pre/post SPPC Intervention scores 1.80 (1.89) to 1.68 (2.36). No SPPC 2.24 (3.04) to 1.65 (2.19). No significant difference (*p* value not reported)
	Coping	Hancock 2018	Brief Cope Inventory, post SPPC intervention score of 5.8 (1.0) for adaptive coping mechanisms vs No SPPC 5.1 (1.1). Not statistically significant overall (*p* = 0.06), however positive reframing (*p* = 0.03) and humor (*p* = 0.04) elements were statistically higher
	Parental health-related quality of life/family functioning	Hancock 2018	PedsQL Family Impact Module. Overall pre/post SPPC intervention 1.1 (10.5) vs No SS − 1.5 (23.7). Early SPPC group communication scores increased by 11.3 points versus 1 point in the standard care group and family relationships scores increased by 5 points versus reduced by 2.6 points in the no SPPC. No statistical differences overall (*p* = 0.68), A positive change in perceived communication [medium effect size of 0.46 (*p* = 0.16)], and family relationship scores [medium effect size of 0.41 (*p* = 0.21)]
System	Cost distribution	Gans 2016	For cardiac patients, the percentage of costs associated with inpatient stays decreased from 71% of costs pre- to 31% post-enrollment, while outpatient costs increased (21% to 50%)

*IQR* interquartile range, *SPPC* subspecialty pediatric palliative care, *LOS* length of stay, *SD* standard deviation, *CICU* cardiac intensive care unit

#### Patient-Level

**Fig. 2:**
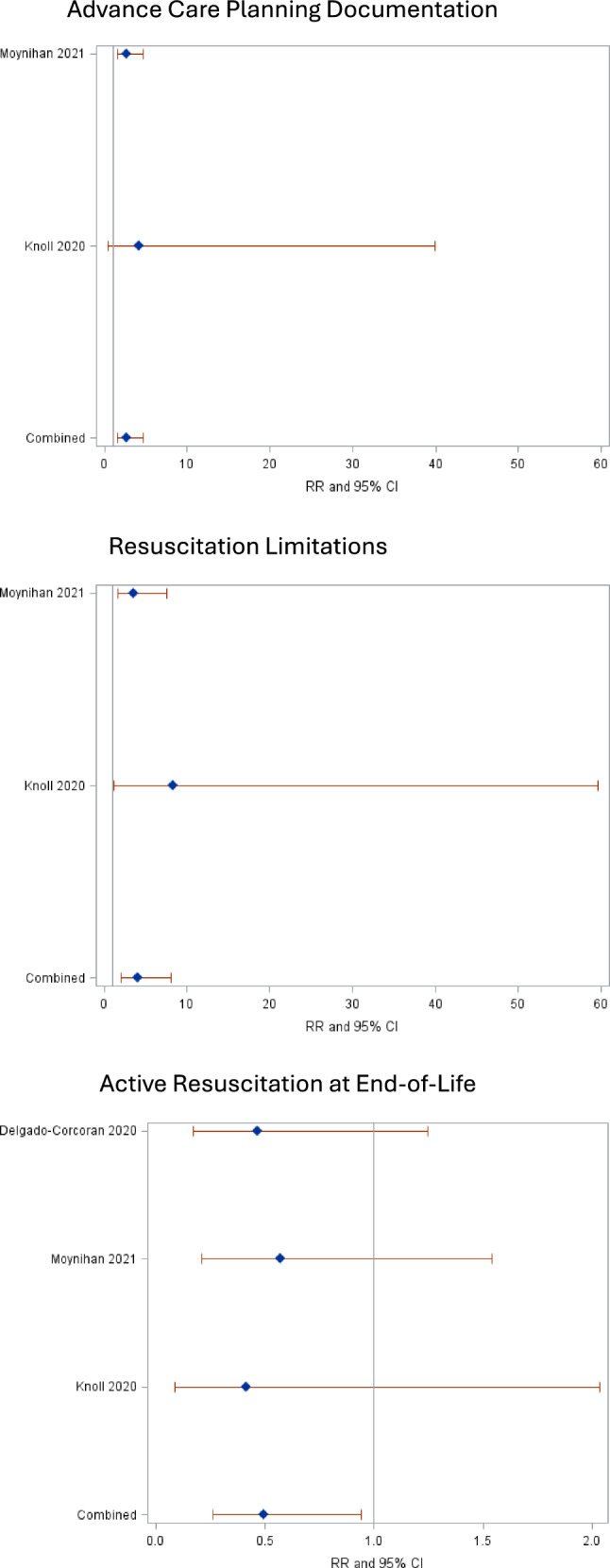
Forest Plots of Relative Risk for Intervention versus Controls for Patient-Level Outcomes. Forest plots demonstrating that combined analyses of relevant studies show statistically increased advanced care planning documentation, increased limitations on resuscitation, and reduced active resuscitation at end-of-life

Length of stay (LOS) was examined in six studies [34–37, 40, 42], survival (four) [35, 36, 40, 41], mode of death (four) [35–37, 41], location of death (three) [35–37], cardiac interventions (surgical vs medical, two studies) [35, 36], ACP (two) [37, 41], resuscitation limitations (two) [37, 41], intensity of medical therapies (one) [37]. Combined risk ratios and forest plots for key outcomes examined in multiple studies are shown (Fig. 2). Patients who received SPPC were 2.7 times more likely to have ACP documented (95%CI 1.6, 4.7, *p* < 0.001), 4.0 times more likely to have resuscitation limits (2.0, 8.1, *p* < 0.001), half as likely to have active resuscitation as the mode of death at end-of-life (EOL) (0.3, 0.9, *p* = 0.032). LOS and survival were not adjusted by disease characteristics in any study. In both, the ‘homogeneity test’ was rejected with discordant direction of associations between retrospective and prospective interventional results.

#### Parent-, Staff- and System-Level

**Fig. 3:**
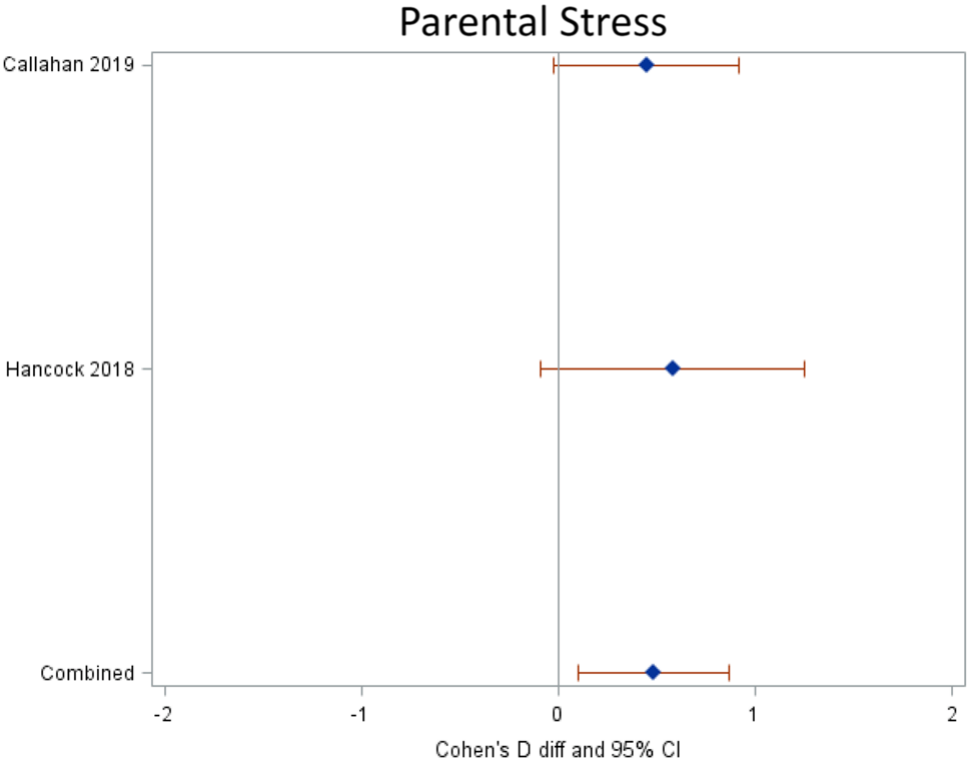
Forest Plot of Relative Risk for Intervention versus Controls for Parental Stress. Forest plot demonstrates statistically significantly improved parental stress based on two relevant studies. Relative risk (RR) estimates provided per study (first author and year named) and combined (pooled estimates, diamond symbol) with 95% confidence intervals (CI) (whiskers) with line at a RR of 1. Active Resuscitation at End-of-Life has a different RR scale

Survey response studies evaluated bereaved parent perceptions of a “good death” [38], parental stress [40, 42], depression [40, 42], anxiety [42], coping [40] and measures of quality of life [40]. For parental stress, receipt of a SPPC intervention improved the scores by almost a half a standard deviation (0.48, 05%CI 0.10, 0.86) pre to post more than controls in this combined estimate (Fig. 3). Only one study examined staff perceptions of quality of dying and death with mixed results overall, but involvement of palliative care increased staff-rated quality of life for the 7 days prior to death [39]. Two examined costs/cost distribution finding a reduction in hospital charges with transition to outpatient care [34, 37].

### Notable Excluded Full-Text Studies

Of 54 articles excluded at full-text, 17 were non-empiric review articles. Of these, eleven specifically discussed the role of SPPC in cardiac patients [5, 10–14, 21–25].

## Discussion

This comprehensive systematic review and meta-analysis summarizes the published literature on the role of SPPC in pediatric heart disease**.** Overall, we identified limited empiric data, generally low in quality, solely from the US, and mostly single center studies with small sample sizes. Even with a paucity of studies, meta-analysis identified benefits of SPPC at both a patient- and family-level for decedents and survivors, with individual studies supporting system-level in-hospital cost reduction and improved staff rating of quality of life prior to death. Pediatric patients who received SPPC were less likely to experience active resuscitation at EOL, and were more likely to have documentation of ACP and resuscitation limits. Additionally, SPPC involvement was found to decrease parental stress. Identified gaps and results offer opportunities for future research and interventions to improve holistic care for children with cardiac disease.

Data supporting benefits of SPPC for children with heart disease mirror results in other populations. The evaluated studies show significant increases in ACP documentation and limits to resuscitation for pediatric patients with AHD who received SPPC, compared to those who did not receive SPPC. This is similar to findings in oncology patients and children with complex chronic conditions who subsequently die as inpatients [18, 43]. One included study evaluated the perspectives of parents of children heart disease who died and found they are more likely to perceive a “good death” experience if they felt prepared, participated in ACP, and reported non-cure-oriented goals-of care [38]. This is consistent with research in parents of children with other complex chronic conditions that shows that parents highly value ACP early in the illness course and that this improves parent-reported EOL outcomes [44].

Of pediatric cardiac patients who died, those who received SPPC were half as likely to experience active resuscitation at EOL. Similar observations were noted in general pediatric patients and children with cancer with SPPC involvement who were both less likely to experience resuscitative events prior to death [18, 45]. Importantly, one included study showed active CPR negatively influenced a family’s perception of a “good death” experience for their child with heart disease [38]. Taken together, core principles of palliative care may promote goal-concordant EOL experiences in this population.

Although EOL and SPPC are often falsely conflated, our meta-analysis demonstrated benefits of SPPC extend beyond decedents. Among parents, parental stress decreased with the receipt of SPPC services [40, 42]. Patients with SVHD have among the highest risk of mortality in AHD, so unsurprisingly this population received increased attention. The randomized control trial of early SPPC intervention for mothers of children with SVHD found decreased maternal anxiety and improved communication and family relationships [40] while parental depression and anxiety did not decrease with the intervention in all-comer neonates with AHD [42]. As part of a quality improvement initiative aiming to improve collaboration between one institution’s Single Ventricle (SV) team and SPPC team, a preliminary survey of Heart Center staff showed that 88% of respondents agreed or strongly agreed that routine involvement of the SPPC team with SV patients has improved the overall psychosocial and/or decision-making support provided to families [46]. While this study lacked a control, it suggests that providers of pediatric cardiac care see benefit from routine involvement for families of children with SVHD.

AHD is a leading cause of disease-related death in US children, and issues around EOL for these patients deserve attention [47]. Despite identifying eleven review articles specifically discussing the role of palliative care in the pediatric cardiac population emphasizing this as a key emerging area and the benefits described, overall SPPC referral rates were low [5, 10–14, 21–25]. Though SPPC involvement has been increasing in children with AHD [37], referral rates have historically been low compared to other pediatric complex conditions [18, 45]. Barriers to SPPC involvement include concerns from pediatric cardiologists of “*undermining parental hope*” and parents perceiving they are “*giving up*” [48, 49]. Notably, in a study evaluating features of SPPC involvement for children with AHD, most families expressed that life prolongation was a priority at the time of initial consultation, which suggests that parents can retain hope and concurrently receive SPPC services [19]. Our evidence suggests that SPPC involvement leads to increased ACP, modes/locations of death that may be more goal-concordant, and improvements in psychosocial support for families of children with AHD. Comparable benefits of SPPC to other patient populations should prompt improved integration of SPPC into the care of pediatric patients with AHD to improve the experience of patients and families as early as immediately after diagnosis, up to end-of-life.

### Limitations and Strengths

There are limitations to consider when interpreting results. Studies are heterogenous in included populations (e.g., CICU decedents vs VAD patients) and the outcome measures examined with no standardized approach to evaluating EOL metrics in pediatric AHD. Overall studies were of low-quality with only 1 RCT, limiting ability to make causal inferences. All studies were conducted in the US in English language, and all were single center apart from one study evaluating parental perspectives from two institutions [38] and a state-based SPPC program implementation study [34], limiting generalizability. Clinicians may be more likely to consult SPPC for children with higher illness severity and/or challenging social situations; we could not assess the impact of these and other potential confounders on mortality and LOS. The fact that benefits of SPPC were observed in retrospective studies where selection bias for referral may exist could suggest larger true benefits. While definitions of SPPC differed between study designs, variable care delivery between hospitals is universal. This review was unable to examine individual interventions or frequency of interventions. Furthermore, patients in the control group may have had effective “primary” palliative care (comprehensive care with a palliative approach delivered by the primary cardiology/intensive care team) which cannot be quantified. When aggregated, statistical findings were stronger for several outcomes despite only two included studies, indicating small sample sizes may also preclude accurate conclusions without meta-analyses. We were unable to include hospice involvement in the meta-analysis due to different definitions of hospice in the included studies, i.e., home with hospice care versus institutional hospice care. This limitation is significant, as dying at home or under hospice conditions is one of the potential outcomes of interest of SPPC involvement described in other pediatric populations [18].

### Future Directions

More work is needed in exploring logistics of optimal SPPC delivery models across different cardiac care environments including when and how subspecialty teams should to be involved versus primary models of delivery. Considering this must span prenatal, intensive care, inpatient, and outpatient involvement, there needs to be parallel efforts to both enhance primary palliative care through training of cardiology and critical care fellows [50, 51] and to consider novel integration approaches [15].

Additional studies are necessary to deepen the body of literature describing the impact of SPPC on the care of children with AHD. Outcomes in retrospective studies primarily centered around SPPC impact on EOL, so an opportunity exists for further research to evaluate the impacts on more holistic benefits extending to survivors or bereavement support after death. Additionally, EOL outcomes studied for pediatric patients are often extrapolated from adult EOL care priorities, such as the location of death being at home. Parental perspectives about preference for location of death has not been studied in pediatric heart disease, and future studies should identify whether preferred location of death was elicited, and if the ultimate location of death was concordant with that preference. There is a dearth of studies evaluating the perspectives of children and adolescents with heart disease receiving SPPC services.

Barriers to more generalizable research through multicenter studies include limitations to ICD-10 coding and cardiac registry data collection. Collaboration with pediatric cardiology and cardiac surgery registries could move the field forward by significantly facilitating larger scale studies [52, 53]. Defining high-quality pediatric EOL care is a priority offering opportunities for standardization of outcomes, which would allow for improved analysis of SPPC impact across institutions [54]. Despite quality metrics for EOL care in oncology [54, 55], no measures are defined for children with AHD, which is a critical gap demanding further research.

### Conclusion

Our systematic review and meta-analysis shows benefits of SPPC for these patients, at both a patient- and family-level with decreased parental stress, less active resuscitation at EOL, and increased ACP documentation and resuscitation limits compared to those without SPPC. Children with AHD are a unique subpopulation of pediatric patients with potentially limited lifespans and high intensity of care through EOL. Overall we identified a paucity of high-quality data studying the influence of SPPC, however findings correlate with literature in other pediatric populations. Results illuminate gaps and future opportunities for research.

## Supplementary Information

Below is the link to the electronic supplementary material.

Supplementary file1 (DOCX 45 KB)

## Data Availability

Systematic review extraction and metanalysis data will be made available on request.
