# Supplementary material for: Impact of Subspecialty Pediatric Palliative Care on Children with Heart Disease; A Systematic Review and Meta-analysis
Source: Pediatr Cardiol. 2024 Jun 22;46(5):1142–56. doi: 10.1007/s00246-024-03535-4 (PMC12021694; doi:10.1007/s00246-024-03535-4)
Supplement: Supplementary file 1 — Supplementary file1 (DOCX 45 KB) [file 246_2024_3535_MOESM1_ESM.docx]

**Impact of Subspecialty Pediatric Palliative Care on Children with Heart Disease; a systematic review and meta-analysis**

James Ting MD*^1^, Kathryn Songer MD*^2^, Valerie Bailey NP, MSN^3^, Chloe Rotman MLIS^4^, Stuart Lipsitz PhD^5^, Abby R. Rosenberg, MD, MS, MA^6,7,8^, Claudia Delgado-Corcoran MD, MPH^2^. Katie M Moynihan MBBS^3.6,9^

^1^ Department of Pediatrics, Johns Hopkins University, Baltimore, MD

^2^ Department of Pediatrics, University of Utah, Salt Lake City, UT

^3^ Department of Cardiology, Boston Children’s Hospital, Boston, MA, USA

^4^ Medical Library, Boston Children’s Hospital, Boston, MA, USA

^5^ Center for Patient Safety, Research, and Practice, Department of General Internal Medicine and Primary Care, Brigham and Women's Hospital, Boston, Massachusetts

^6^ Department of Pediatrics, Harvard Medical School, Boston, MA, USA

^7^ Department of Psychosocial Oncology and Palliative Care; Dana-Farber Cancer Institute, Boston, MA, USA

^8^ Department of Pediatrics, Boston Children’s Hospital, Boston, MA, USA

^9^ Children’s Hospital at Westmead Clinical School, Faculty of Medicine and Health, The University of Sydney, Sydney, NSW, Australia.

* Contributed equally as co-first authors

**Corresponding author and to request reprints:** Katie M. Moynihan, MBBS, FRACP, FCICM Department of Cardiology, MS BCH3215. Boston Children’s Hospital, 300 Longwood Ave, 02115, Boston, MA, USA. T: 617-355-4023/F: 617-730-7548. Email Katie.Moynihan@cardio.chboston.org.

**Supplemental Table 1:** Summary risk bias by papers

| Study ID | Data source | Study funding Y/N and sources if Y | 1. Study Participation: | 2. Study Attrition | 3. Prognostic/ Predictive Factor | 4. Outcome Measurement | 5. Confounder Measurement | 6. Statistical Analysis and Reporting |
| --- | --- | --- | --- | --- | --- | --- | --- | --- |
| Gans 2016* | California Department of Health Care Services (DHCS), including paid Medicaid claims and PFC enrollment data | California Department of Health Care Services (contract number 09-86215) | High Bias = Low Quality | High Bias = Low Quality | Moderate Bias/Quality | Low Bias = High Quality | High Bias = Low Quality | High Bias = Low Quality |
| Delgado-Corcoran 2021 | Institutional EMR | National Institute of Nursing Research of the National Institutes of Health | High Bias = Low Quality | Moderate Bias/Quality | Low Bias = High Quality | Moderate Bias/Quality | High Bias = Low Quality | High Bias = Low Quality |
| Moynihan 2021 | Institutional EMR | Rochelle E. Rose Cardiac ICU Research Fund | Moderate Bias/Quality | Moderate Bias/Quality | Low Bias = High Quality | Moderate Bias/Quality | High Bias = Low Quality | High Bias = Low Quality |
| Bailey 2022 | Institutional EMR and survey of CICU staff. | New England Congenital Cardiology Research Foundation (NECCRF). | Moderate Bias/Quality | Moderate Bias/Quality | Low Bias = High Quality | Moderate Bias/Quality | High Bias = Low Quality | High Bias = Low Quality |
| Delgado-Corcoran 2020 | Institutional EMR | National Institute of Nursing Research; she received support for article research from the National Institutes of Health under award number T32NR013456 (m-PIs: Ellington  and Mooney); and she disclosed government work. | Moderate Bias/Quality | Moderate Bias/Quality | Low Bias = High Quality | Moderate Bias/Quality | High Bias = Low Quality | High Bias = Low Quality |
| Hancock 2018 | Institutional EMR and survey responses | Internal funding was provided through the Michigan Congenital Heart Outcomes Research and Discovery multidisciplinary programme in the form of the Greise Hutchinson Woodson Pilot Grant Award | Low Bias = High Quality | Moderate Bias/Quality | Low Bias = High Quality | Moderate Bias/Quality | Moderate Bias/Quality | High Bias = Low Quality |
| Moynihan 2022 | Bereaved parent survey and institutional EMR | Grousbeck Fazzarelli Fund for Cardiology Research | High Bias = Low Quality | High Bias = Low Quality | Low Bias = High Quality | Moderate Bias/Quality | High Bias = Low Quality | High Bias = Low Quality |
| Knoll 2020 | Institutional EMR | None | Moderate Bias/Quality | High Bias = Low Quality | Low Bias = High Quality | Moderate Bias/Quality | High Bias = Low Quality | High Bias = Low Quality |
| Callahan 2019 | Institutional EMR and parental survey responses 2 months apart | None mentioned but Neonatal Comfort Care Program team are acknowledged for their participation in and support of this study. | Moderate Bias/Quality | Moderate Bias/Quality | Low Bias = High Quality | Moderate Bias/Quality | Moderate Bias/Quality | High Bias = Low Quality |

* Conflict reported: Two of the authors work for the agency that supported this work through a contract for evaluation

**Supplemental Table 2**: PRISMA Checklist

| Section/Topic | # | Checklist Item | Reported on Page # |
| --- | --- | --- | --- |
| **TITLE** | | | |
| Title | 1 | Identify the report as a systematic review, meta-analysis, or both. | 1 |
| **ABSTRACT** | | | |
| Structured summary | 2 | Provide a structured summary including, as applicable: background; objectives; data sources; study eligibility criteria, participants, and interventions; study appraisal and synthesis methods; results; limitations; conclusions and implications of key findings; systematic review registration number. | 2 |
| **INTRODUCTION** | | | |
| Rationale | 3 | Describe the rationale for the review in the context of what is already known. | 3 |
| Objectives | 4 | Provide an explicit statement of questions being addressed with reference to participants, interventions, comparisons, outcomes, and study design (PICOS). | 3 |
| **METHODS** | | | |
| Protocol and registration | 5 | Indicate if a review protocol exists, if and where it can be accessed (e.g., Web address), and, if available, provide registration information including registration number. | 4 |
| Eligibility criteria | 6 | Specify study characteristics (e.g., PICOS, length of follow-up) and report characteristics (e.g., years considered, language, publication status) used as criteria for eligibility, giving rationale. | 4 |
| Information sources | 7 | Describe all information sources (e.g., databases with dates of coverage, contact with study authors to identify additional studies) in the search and date last searched. | 4 |
| Search | 8 | Present full electronic search strategy for at least one database, including any limits used, such that it could be repeated. | 4 |
| Study selection | 9 | State the process for selecting studies (i.e., screening, eligibility, included in systematic review, and, if applicable, included in the meta-analysis). | 5 |
| Data collection process | 10 | Describe method of data extraction from reports (e.g., piloted forms, independently, in duplicate) and any processes for obtaining and confirming data from investigators. | 5 |
| Data items | 11 | List and define all variables for which data were sought (e.g., PICOS, funding sources) and any assumptions and simplifications made. | 5 |
| Risk of bias in individual studies | 12 | Describe methods used for assessing risk of bias of individual studies (including specification of whether this was done at the study or outcome level), and how this information is to be used in any data synthesis. | 5 |
| Summary measures | 13 | State the principal summary measures (e.g., risk ratio, difference in means). | 5-6 |
| Synthesis of results | 14 | Describe the methods of handling data and combining results of studies, if done, including measures of consistency (e.g., I2) for each meta-analysis. | 5-6 |
| Risk of bias across studies | 15 | Specify any assessment of risk of bias that may affect the cumulative evidence (e.g., publication bias, selective reporting within studies). | 5-6 |
| Additional analyses | 16 | Describe methods of additional analyses (e.g., sensitivity or subgroup analyses, meta-regression), if done, indicating which were pre-specified. | 5-6 |
| **RESULTS** | | | |
| Study selection | 17 | Give numbers of studies screened, assessed for eligibility, and included in the review, with reasons for exclusions at each stage, ideally with a flow diagram. | 7 |
| Study characteristics | 18 | For each study, present characteristics for which data were extracted (e.g., study size, PICOS, follow-up period) and provide the citations. | 7 |
| Risk of bias within studies | 19 | Present data on risk of bias of each study and, if available, any outcome-level assessment (see Item 12). | 7 |
| Results of individual studies | 20 | For all outcomes considered (benefits or harms), present, for each study: (a) simple summary data for each intervention group and (b) effect estimates and confidence intervals, ideally with a forest plot. | 7 |
| Synthesis of results | 21 | Present results of each meta-analysis done, including confidence intervals and measures of consistency. | 7-8 |
| Risk of bias across studies | 22 | Present results of any assessment of risk of bias across studies (see Item 15). | 7 |
| Additional analysis | 23 | Give results of additional analyses, if done (e.g., sensitivity or subgroup analyses, meta-regression [see Item 16]). | 7-8 |
| **DISCUSSION** | | | |
| Summary of evidence | 24 | Summarize the main findings including the strength of evidence for each main outcome; consider their relevance to key groups (e.g., health care providers, users, and policy makers). | 8-10 |
| Limitations | 25 | Discuss limitations at study and outcome level (e.g., risk of bias), and at review level (e.g., incomplete retrieval of identified research, reporting bias). | 10-11 |
| Conclusions | 26 | Provide a general interpretation of the results in the context of other evidence, and implications for future research. | 11-12 |
| **FUNDING** | | | |
| Funding | 27 | Describe sources of funding for the systematic review and other support (e.g., supply of data); role of funders for the systematic review. | 1 |

**Supplemental Table 3** – Search strategy

| PubMed | (Pediatrics[mesh] OR pediatric*[tiab] OR "Infant, newborn"[mesh] OR newborn*[tiab] OR neonat*[tiab] OR Infant[mesh] OR infant*[tiab] OR toddler*[tiab] OR baby[tiab] OR babies[tiab] OR "Child, preschool"[mesh] OR "school age*"[tiab] OR "preschool age*"[tiab] OR "pre school age"[tiab] OR child[mesh] OR child*[tiab] OR adolescent[mesh] OR adolescen*[tiab] OR teen*[tiab] OR youth*[tiab]) AND ("palliative medicine"[mesh] OR "palliative medicine"[tiab] OR "supportive medicine"[tiab] OR "hospice and palliative care nursing"[mesh] OR "palliative nursing"[tiab] OR hospices[mesh] OR hospice*[tiab] OR "hospice care"[mesh] OR "bereavement care"[tiab] OR "bereavement support"[tiab] "palliative care"[mesh] OR palliative[tiab] OR "Supportive Care"[tiab] OR "Supportive treatment*"[tiab] OR "supportive therap*"[tiab] OR "compassionate care"[tiab] OR "terminal care"[mesh] OR "terminal care"[tiab] OR "end of life care"[tiab]) AND ("Quality of Life"[mesh] OR "Psychological Well-Being"[mesh] OR "Personal Satisfaction"[mesh] OR "patient satisfaction"[mesh] OR "Job Satisfaction"[mesh] OR pain[mesh] OR "Stress, Psychological"[mesh] OR "Outcome Assessment, Health Care"[mesh] OR "Patient Outcome Assessment"[mesh] OR "Treatment Outcome"[mesh] OR "Physician-Patient Relations"[mesh] OR "Health Personnel"[mesh] OR Physicians[mesh] OR Nurses[mesh] OR "Delivery of Health Care"[mesh] OR "Intensive Care Units, Pediatric"[mesh] OR "quality of life"[Title/Abstract] OR "life quality"[Title/Abstract] OR hrqol[Title/Abstract] OR "wellbeing" OR "Well Being"[Title/Abstract] OR "ill being"[Title/Abstract] OR satisfaction[Title/Abstract] OR satisfied[Title/Abstract] OR "Job Satisfaction"[Title/Abstract] OR "work satisfaction"[Title/Abstract] OR pain[Title/Abstract] OR suffer*[Title/Abstract] OR stress*[Title/Abstract] OR distress*[Title/Abstract] OR outcome*[Title/Abstract] OR OR[Title/Abstract] OR "Physician-Patient Relation*"[Title/Abstract] OR "Doctor Patient Relation*"[Title/Abstract] OR "health personnel"[Title/Abstract] OR "medical personnel"[Title/Abstract] OR "Health Care Provider*"[Title/Abstract] OR "Healthcare Provider*"[Title/Abstract] OR "medical staff"[Title/Abstract] OR "health care staff"[Title/Abstract] OR "healthcare staff"[Title/Abstract] OR "Health Care Professional*"[Title/Abstract] OR "Healthcare Professional*"[Title/Abstract] OR "medical professional*"[Title/Abstract] OR physician*[Title/Abstract] OR doctor*[Title/Abstract] OR nurse*[Title/Abstract] OR "Delivery of Health Care"[Title/Abstract] OR "Delivery of Healthcare"[Title/Abstract] OR "Health Care Delivery"[Title/Abstract] OR "Healthcare Delivery"[Title/Abstract] OR "Health Care System*"[Title/Abstract] OR "Healthcare System*"[Title/Abstract] OR "Pediatric Intensive Care Unit*"[Title/Abstract] OR PICU[Title/Abstract] OR "cardiac intensive care unit*"[Title/Abstract] OR CICU OR[Title/Abstract]) AND ("Heart Defects, Congenital"[mesh] OR "congenital heart disease*"[tiab] OR CHD[tiab] OR "congenital heart defect*"[tiab] OR "congenital heart malformation*"[tiab] OR "congenital heart abnormalit*"[tiab] OR "congenital cardiac disease*"[tiab] OR "congenital cardiac defect*"[tiab] OR "congenital cardiac malformation*"[tiab] OR "congenital cardiac abnormalit*"[tiab] OR "congenital cardiovascular anomal*"[tiab] OR "congenital cardiovascular malformation*"[tiab] OR "congenital cardiovascular defect*"[tiab] OR "Aortic Coarctation"[mesh] OR "Aortic Coarctation*"[tiab] OR "Coarctation of the Aorta"[tiab] OR "Coarctation of Aorta"[tiab] OR "Aorta Coarctation*"[tiab] OR "Aorta Dominant Coarctation"[tiab:~0] OR "Aortico-Ventricular Tunnel"[mesh] OR "Aortico-Ventricular Tunnel*"[tiab] OR "Aortic Right Ventricular Tunnel*"[tiab] OR "Aortic Left Ventricular Tunnel*"[tiab] OR "Aortoventricular Tunnel*"[tiab] OR "Aorto Ventricular Tunnel*"[tiab] OR "Aorticoventricular Tunnel*"[tiab] OR "Aortic Ventricular Tunnel*"[tiab] OR "Aortico Left Ventricular Tunnel*"[tiab] OR "Aorto Left Ventricular Tunnel*"[tiab] OR "Aortico Right Ventricular Tunnel*"[tiab] OR "Aorto Right Ventricular Tunnel*"[tiab] OR "Arrhythmogenic Right Ventricular Dysplasia"[mesh] OR "Arrhythmogenic Right Ventricular Dysplasia"[tiab] OR "Arrhythmogenic Right Ventricular Cardiomyopathy Dysplasia"[tiab] OR "ARVD-C"[tiab] OR "Arrhythmogenic Right Ventricular Cardiomyopathy"[tiab] OR "Arrhythmogenic Right Ventricular Dysplasia-Cardiomyopathy"[tiab] OR "Bicuspid Aortic Valve Disease"[mesh] OR "Bicuspid Aortic Valve Disease*"[tiab] OR "Aortic Valve Disease 1"[tiab] OR "Cor Triatriatum"[mesh] OR "Cor Triatriatum"[tiab] OR "Subdivided Left Atrium*"[tiab] OR "Triatrial Heart*"[tiab] OR "Coronary Vessel Anomalies"[mesh] OR "Coronary Vessel Anomal*"[tiab] OR "Anomalous Left Coronary Artery"[mesh] OR "Anomalous Left Coronary Artery"[tiab] OR "Bland White Garland Syndrome"[mesh] OR "Bland White Garland Syndrome"[tiab] OR ALCAPA[tiab] OR "Myocardial Bridging"[mesh] OR "Myocardial Bridging*"[tiab] OR "Myocardial Bridgings"[tiab] OR "Crisscross Heart"[mesh] OR "Crisscross Heart*"[tiab] OR "Criss cross Heart*"[tiab] OR Dextrocardia[mesh] OR Dextrocardia*[tiab] OR "Ductus Arteriosus, Patent"[mesh] OR "Patent Ductus Arteriosus"[tiab] OR "Patency of the Ductus Arteriosus"[tiab] OR "Ebstein Anomaly"[mesh] OR "Ebstein Anomaly"[tiab] OR "Ebstein's Malformation"[tiab] OR "Ebstein Malformation"[tiab] OR "Ebsteins Malformation"[tiab] OR "Ebstein's Anomaly"[tiab] OR "Ebsteins Anomaly"[tiab] OR "Ectopia Cordis"[mesh] OR "Ectopia Cordis"[tiab] OR "Eisenmenger Complex"[mesh] OR "Eisenmenger Complex"[tiab] OR "Eisenmenger's Complex"[tiab] OR "Eisenmengers Complex"[tiab] OR "Eisenmenger's Syndrome"[tiab] OR "Eisenmengers Syndrome"[tiab] OR "Eisenmenger Syndrome"[tiab] OR "heart septal defects"[mesh] OR "septal defect*"[tiab] OR "Truncus Arteriosus, Persistent"[mesh] OR "Persistent Truncus Arteriosus"[tiab] OR "Endocardial Cushion Defects"[mesh] OR "Endocardial Cushion Defect*"[tiab] OR "Persistent Common Atrioventricular Canal"[tiab] OR "Heart Septal Defects, Atrial"[mesh] OR "Persistent Ostium Primum"[tiab] OR "Foramen Ovale, Patent"[mesh] OR "Patent Oval Foramen"[tiab] OR "Patent Foramen Ovale"[tiab] OR "Lutembacher Syndrome"[mesh] OR "Lutembacher Syndrome"[tiab:~0] OR "Lutembacher's Syndrome"[tiab] OR "Lutembachers Syndrome"[tiab:~0] OR "Double Outlet Right Ventricle"[mesh] OR "Double Outlet Right Ventricle*"[tiab] OR "Taussig Bing Anomaly"[tiab] OR "Heterotaxy Syndrome"[mesh] OR "Heterotaxy Syndrome*"[tiab] OR "Visceral Heterotax*"[tiab] OR "Situs Ambiguus"[tiab] OR "Polysplenia Syndrome*"[tiab] OR "Left Atrial Isomerism with Polysplenia"[tiab:~0] OR "Right Atrial Isomerism*"[tiab] OR "Asplenia Syndrome*"[tiab] OR "Right Atrial Isomerism with Asplenia"[tiab:~0] OR "Asplenia with Cardiovascular Anomalies"[tiab:~0] OR "Ivemark Syndrome"[tiab] OR "Left Atrial Isomerism*"[tiab] OR "Hypoplastic Left Heart Syndrome"[mesh] OR "Hypoplastic Left Heart Syndrome"[tiab] OR "Left Heart Hypoplasia Syndrome"[tiab] OR "Isolated Noncompaction of the Ventricular Myocardium"[mesh] OR "Isolated Noncompaction of the Ventricular Myocardium"[tiab] OR "Isolated Non-compaction of the Ventricular Myocardium"[tiab] OR "Noncompaction of the Left Ventricular Myocardium"[tiab] OR "LEOPARD Syndrome"[mesh] OR "LEOPARD Syndrome"[tiab] OR "Lentiginosis Cardiomyopathic"[tiab:~0] OR "Progressive Cardiomyopathic Lentiginos*"[tiab] OR "Cardio Cutaneous Syndrome"[tiab] OR "Cardiomyopathic Lentiginos*"[tiab] OR Levocardia[mesh] OR Levocardia[tiab] OR "Quadricuspid Aortic Valve"[mesh] OR "Quadricuspid Aortic Valve*"[tiab] OR "Quadricuspid Aortic Valvular Disease"[tiab:~0] OR "Tetralogy of Fallot"[mesh] OR "Tetralogy of Fallot"[tiab] OR "Fallot Tetralogy"[tiab] OR "Fallot's Tetralogy"[tiab] OR "Fallots Tetralogy"[tiab] OR "Transposition of Great Vessels"[mesh] OR "Transposition of Great Vessels"[tiab] OR "Great Vessels Transposition*"[tiab] OR "Transposition of the Great Arteries"[tiab] OR "Great Arteries Transposition*"[tiab] OR "Congenitally Corrected Transposition of the Great Arteries"[mesh] OR "Congenitally Corrected Transposition*"[tiab] OR "Tricuspid Atresia"[mesh] OR "Tricuspid Atresia*"[tiab] OR "Tricuspid Valve Atresia*"[tiab] OR "Trilogy of Fallot"[mesh] OR "Trilogy of Fallot"[tiab] OR "Fallot Trilogy"[tiab] OR "Fallot's Trilogy"[tiab] OR "Fallots Trilogy"[tiab:~0] OR "Univentricular Heart"[mesh] OR "Univentricular Heart*"[tiab] OR "Complex Single Ventricle*"[tiab] OR "Congenital heart block"[Supplementary Concept] "congenital heart block"[tiab] OR "Atrioventricular Block"[mesh] OR "Atrioventricular Block"[tiab] OR "congenital cardiac block"[tiab:~0] OR "congenital cardial block"[tiab:~0] OR "Supravalvar Aortic Stenosis Syndrome"[tiab] OR "Hypercalcemia Supravalvar Aortic Stenosis"[tiab:~0] OR "Pulmonary Arterial Hypertension"[mesh] OR "Pulmonary Arterial Hypertension"[tiab] OR "Pulmonary Artery Hypertension"[tiab] OR Cardiology[mesh] OR cardiology[tiab] OR "Heart Diseases"[mesh] OR "Heart Disease*"[tiab] OR "heart disorder"[tiab] OR "cardiac disease*"[tiab] OR "cardiac disorder*"[tiab] OR "Arrhythmias, Cardiac"[mesh] OR arrhythmia*[tiab] OR dysrhythmia*[tiab] OR "Carcinoid Heart Disease"[mesh] OR "Carcinoid Heart Disease*"[tiab] OR "Cardiac Conduction System Disease"[mesh] OR "cardiac conduction"[tiab] OR "Cardiac Output, High"[mesh] OR "high cardiac output"[tiab] OR "Cardiac Output, Low"[mesh] OR "low cardiac output"[tiab] OR "Cardiac Tamponade"[mesh] OR "Cardiac Tamponade*"[tiab] OR "Pericardial Tamponade*"[tiab] OR Cardiomegaly[mesh] OR Cardiomegaly[tiab] OR "Enlarged Heart*"[tiab] OR "Cardiac Hypertroph*"[tiab] OR "Heart Hypertroph*"[tiab] OR Cardiomyopathies[mesh] OR Cardiomyopath*[tiab] OR Myocardiopath*[tiab] OR "Myocardial Disease*"[tiab] OR Cardiotoxicity[mesh] OR Cardiotoxicit*[tiab] OR "Cardiac Toxicit*"[tiab] OR Endocarditis[mesh] OR Endocarditis[tiab] OR "Heart Valve Diseases"[mesh] OR "Heart Valve Disease*"[tiab] OR "valve disease*"[tiab] OR "valve insufficiency"[tiab] OR "valve stenosis"[tiab] OR "valve prolapse"[tiab] OR "valve atresia"[tiab] OR "pulmonary atresia"[mesh] OR "pulmonary atresia"[tiab] OR "Myocardial Ischemia"[mesh] OR "Myocardial Ischemia*"[tiab] OR "Ischemic Heart Disease*"[tiab] OR "Myocardial Stunning"[mesh] OR "Myocardial Stunning"[tiab] OR "Myocardial Hibernation"[tiab] OR "Stunned Myocardium"[tiab] OR "Pericardial Effusion"[mesh] OR "Pericardial Effusion*"[tiab] OR Hemopericardium[tiab] OR Chylopericardium[tiab] OR Pericarditis[mesh] OR Pericarditis[tiab] OR Pleuropericarditis[tiab] OR Pneumopericardium[mesh] OR Pneumopericardium[tiab] OR "Post-Cardiac Arrest Syndrome"[mesh] OR "Post-Cardiac Arrest Syndrome*"[tiab] OR "Postresuscitation Disease*"[tiab] OR "Postcardiac Arrest Syndrome*"[tiab] OR "Postpericardiotomy Syndrome"[mesh] OR "Postpericardiotomy Syndrome*"[tiab] OR "Postcommissurotomy Syndrome*"[tiab] OR "Pulmonary Heart Disease"[mesh] OR "Pulmonary Heart Disease*"[tiab] OR "Rheumatic Heart Disease"[mesh] OR "Rheumatic Heart Disease*"[tiab] OR "Bouillaud Disease"[tiab] OR "Bouillaud's Disease"[tiab] OR "Bouillauds Disease"[tiab:~0] OR "Ventricular Dysfunction"[mesh] OR "Ventricular Dysfunction*"[tiab] OR "Ventricular Outflow Obstruction"[mesh] OR "Ventricular Outflow Obstruction*"[tiab] OR "Arrhythmia, Sinus"[mesh] OR "Sinus Arrhythmia*"[tiab] OR "Sinoatrial Arrhythmia*"[tiab] OR "Sick Sinus Syndrome"[mesh] OR "Sick Sinus Syndrome"[tiab] OR "Sick Sinus Node Syndrome"[tiab] OR "Sinus Node Dysfunction"[tiab] OR "Sinus Node Disease*"[tiab] OR "Sinus Arrest, Cardiac"[mesh] OR "Cardiac Sinus Arrest*"[tiab] OR "Cardiac Sinus Pause*"[tiab] OR "Atrial Fibrillation"[mesh] OR "Atrial Fibrillation*"[tiab] OR "Auricular Fibrillation*"[tiab] OR "Atrial Flutter"[mesh] OR "Atrial Flutter*"[tiab] OR "Auricular Flutter*"[tiab] OR Bradycardia[mesh] OR Bradycardia*[tiab] OR Bradyarrhythmia*[tiab] OR "Brugada Syndrome"[mesh] OR "Brugada Syndrome"[tiab] OR "Sudden Unexplained Death Syndrome"[tiab] OR "Sudden Unexplained Nocturnal Death Syndrome"[tiab] OR "Brugada ECG Pattern"[tiab] OR "Brugada Type ECG Pattern"[tiab] OR "Cardiac Complexes, Premature"[mesh] OR "Premature Beats*"[tiab] OR Extrasystole[tiab] OR "Premature Cardiac Complex"[tiab:~0] OR "Ectopic Heartbeat*"[tiab] OR "Commotio Cordis"[mesh] OR "Commotio Cordis"[tiab] OR "Cardiac Concussion*"[tiab] OR "Heart Block"[mesh] OR "Heart Block*"[tiab] OR "Auriculo-Ventricular Dissociation*"[tiab] OR "Atrioventricular Dissociation*"[tiab] OR "A V Dissociation*"[tiab] OR "Long QT Syndrome"[mesh] OR "Long QT Syndrome"[tiab] OR Parasystole[mesh] OR Parasystole*[tiab] OR "Pre-Excitation Syndromes"[mesh] OR "Pre-Excitation Syndrome*"[tiab] OR "Preexcitation Syndrome*"[tiab] OR Tachycardia[mesh] OR Tachycardia*[tiab] OR Tachyarrhythmia*[tiab] OR "Ventricular Fibrillation"[mesh] OR "Ventricular Fibrillation*"[tiab] OR "Ventricular Flutter"[mesh] OR "Ventricular Flutter*"[tiab] OR "Cardiomyopathy, Dilated"[mesh] OR "Dilated Cardiomyopath*"[tiab] OR "Idiopathic Cardiomyopath*"[tiab] OR "Congestive Cardiomyopath*"[tiab] OR "Hypertrophy, Left Ventricular"[mesh] OR "ventricular hypertroph*"[tiab] OR "Hypertrophy, Right Ventricular"[mesh] OR "Hypertrophic Cardiomyopath*"[tiab] OR "Heart Failure"[mesh] OR "Heart Failure"[tiab] OR "Cardio-Renal Syndrome"[mesh] OR "Cardio-Renal Syndrome*"[tiab] OR "Reno-Cardiac Syndrome*"[tiab] OR "Renocardiac Syndrome*"[tiab] OR "Cardiorenal Syndrome*"[tiab] OR "Coronary Disease"[mesh] OR "Coronary Disease*"[tiab] OR "Coronary Aneurysm"[mesh] OR "Coronary Aneurysm*"[tiab] OR "Coronary Artery Disease"[mesh] OR "Coronary Artery Disease*"[tiab] OR "Left Main Disease*"[tiab] OR "Coronary Arterioscleros*"[tiab] OR "Coronary Atheroscleros*"[tiab] OR "Coronary Occlusion"[mesh] OR "Coronary Occlusion*"[tiab] OR "Coronary Stenosis"[mesh] OR "Coronary Stenos*"[tiab] OR "Coronary Artery Stenos*"[tiab] OR "Coronary Restenosis"[mesh] OR "Coronary Restenos*"[tiab] OR "Coronary-Subclavian Steal Syndrome"[mesh] OR "Coronary-Subclavian Steal Syndrome*"[tiab] OR "Coronary Thrombosis"[mesh] OR "Coronary Thrombos*"[tiab] OR "Coronary Vasospasm"[mesh] OR "Coronary Vasospasm*"[tiab] OR "Coronary Artery Spasm*"[tiab] OR "Coronary Spasm*"[tiab] OR "Myocardial Infarction"[mesh] OR "Myocardial Infarct**"[tiab] OR "Cardiovascular Stroke*"[tiab] OR "Heart Attack*"[tiab] OR "Myocardial Reperfusion Injury"[mesh] OR "Myocardial Reperfusion Injur*"[tiab] OR "Myocardial Ischemic Reperfusion Injur*"[tiab]) AND ((english[Language] OR spanish[language]) AND ("2000"[Date - Publication] : "3000"[Date - Publication])) |
| --- | --- |
| EMBASE | ('pediatrics'/exp OR 'newborn'/exp OR 'infant'/exp OR 'toddler'/exp OR 'child'/exp OR 'adolescent'/exp OR pediatric*:ab,ti OR newborn*:ab,ti OR neonat*:ab,ti OR infant*:ab,ti OR toddler*:ab,ti OR baby:ab,ti OR babies:ab,ti OR 'school age*':ab,ti OR 'preschool age*':ab,ti OR 'pre school age':ab,ti OR child*:ab,ti OR adolescen*:ab,ti OR teen*:ab,ti OR youth*:ab,ti) AND ('palliative therapy'/exp OR 'palliative nursing'/exp OR 'hospice nursing'/exp OR 'hospice'/exp OR 'hospice care'/exp OR 'terminal care'/exp OR 'palliative medicine':ab,ti OR 'supportive medicine':ab,ti OR 'palliative nursing':ab,ti OR hospice*:ab,ti OR 'bereavement care':ab,ti OR 'bereavement support':ab,ti OR palliative:ab,ti OR 'supportive care':ab,ti OR 'supportive treatment*':ab,ti OR 'supportive therap*':ab,ti OR 'compassionate care':ab,ti OR 'terminal care':ab,ti OR 'end of life care':ab,ti) AND ('quality of life'/exp OR 'wellbeing'/exp OR 'emotional well-being'/exp OR 'satisfaction'/exp OR 'patient satisfaction'/exp OR 'job satisfaction'/exp OR 'pain'/exp OR 'mental stress'/exp OR 'outcome assessment'/exp OR 'treatment outcome'/exp OR 'doctor patient relationship'/exp OR 'health care personnel'/exp OR 'physician'/exp OR 'nurse'/exp OR 'health care delivery'/exp OR 'pediatric intensive care unit'/exp OR 'quality of life':ab,ti OR 'life quality':ab,ti OR hrqol:ab,ti OR wellbeing:ab,ti OR 'well being':ab,ti OR 'ill being':ab,ti OR satisfaction:ab,ti OR satisfied:ab,ti OR 'job satisfaction':ab,ti OR 'work satisfaction':ab,ti OR pain:ab,ti OR suffer*:ab,ti OR stress*:ab,ti OR distress*:ab,ti OR outcome*:ab,ti OR 'physician-patient relation*':ab,ti OR 'doctor patient relation*':ab,ti OR 'health personnel':ab,ti OR 'medical personnel':ab,ti OR 'health care provider*':ab,ti OR 'healthcare provider*':ab,ti OR 'medical staff':ab,ti OR 'health care staff':ab,ti OR 'healthcare staff':ab,ti OR 'health care professional*':ab,ti OR 'healthcare professional*':ab,ti OR 'medical professional*':ab,ti OR physician*:ab,ti OR doctor*:ab,ti OR nurse*:ab,ti OR 'delivery of health care':ab,ti OR 'delivery of healthcare':ab,ti OR 'health care delivery':ab,ti OR 'healthcare delivery':ab,ti OR 'health care system*':ab,ti OR 'healthcare system*':ab,ti OR 'pediatric intensive care unit*':ab,ti OR picu:ab,ti OR 'cardiac intensive care unit*':ab,ti OR cicu:ab,ti) AND ('congenital heart malformation'/exp OR 'congenital heart disease'/exp OR 'aortic coarctation'/exp OR 'aortico-ventricular tunnel'/exp OR 'heart right ventricle dysplasia'/exp OR 'bicuspid aortic valve'/exp OR 'cor triatriatum'/exp OR 'coronary vessel malformation'/exp OR 'coronary artery anomaly'/exp OR 'bland white garland syndrome'/exp OR 'myocardial bridging'/exp OR 'criss cross atrioventricular relationship'/exp OR 'dextrocardia'/exp OR 'patent ductus arteriosus'/exp OR 'ebstein anomaly'/exp OR 'ectopia cordis'/exp OR 'eisenmenger complex'/exp OR 'heart septum defect'/exp OR 'heart atrium septum defect'/exp OR 'patent foramen ovale'/exp OR 'heart right ventricle double outlet'/exp OR 'heterotaxy syndrome'/exp OR 'hypoplastic left heart syndrome'/exp OR 'ventricular noncompaction'/exp OR 'leopard syndrome'/exp OR 'levocardia'/exp OR 'quadricuspid aortic valve'/exp OR 'fallot tetrology' OR 'great vessels transposition'/exp OR 'congenitally corrected transposition of the great arteries'/exp OR 'tricuspid valve atresia'/exp OR 'heart single ventricle'/exp OR 'congenital heart block'/exp OR 'atrioventricular block'/exp OR 'cardiology'/exp OR 'heart disease'/exp OR 'heart arrhythmia'/exp OR 'carcinoid heart disease'/exp OR 'heart muscle conduction disturbance'/exp OR 'high output heart failure'/exp OR 'forward heart failure'/exp OR 'heart tamponade'/exp OR 'cardiomegaly'/exp OR 'cardiomyopathy'/exp OR 'cardiotoxicity'/exp OR 'endocarditis'/exp OR 'valvular heart disease'/exp OR 'pulmonary valve atresia'/exp OR 'heart muscle ischemia'/exp OR 'stunned heart muscle'/exp OR 'pericardial effusion'/exp OR 'pericarditis'/exp OR 'pneumopericardium'/exp OR 'post-cardiac arrest syndrome'/exp OR 'postpericardiotomy syndrome'/exp OR 'cor pulmonale'/exp OR 'rheumatic heart disease'/exp OR 'heart ventricle function'/exp OR 'heart outflow tract obstruction'/exp OR 'sinus arrhythmia'/exp OR 'sick sinus syndrome'/exp OR 'sinus arrest'/exp OR 'atrial fibrillation'/exp OR 'heart atrium flutter'/exp OR 'bradycardia'/exp OR 'brugada syndrome'/exp OR 'extrasystole'/exp OR 'commotio cordis'/exp OR 'heart block'/exp OR 'long qt syndrome'/exp OR 'parasystole'/exp OR 'heart preexcitation'/exp OR 'tachycardia'/exp OR 'heart ventricle fibrillation'/exp OR 'heart ventricle flutter'/exp OR 'dilated cardiomyopathy'/exp OR 'left ventricular hypertrophy'/exp OR 'right ventricular hypertrophy'/exp OR 'heart failure'/exp OR 'cardiorenal syndrome'/exp OR 'coronary artery aneurysm'/exp OR 'coronary artery disease'/exp OR 'coronary occlusion'/exp OR 'coronary stenosis'/exp OR 'coronary restenosis'/exp OR 'coronary subclavian steal syndrome'/exp OR 'coronary artery thrombosis'/exp OR 'coronary artery spasm'/exp OR 'heart infarction'/exp OR 'myocardial ischemia reperfusion injury'/exp OR 'congenital heart disease':ab,ti OR 'congenital heart diseases':ab,ti OR chd:ab,ti OR 'congenital heart defect':ab,ti OR 'congenital heart defects':ab,ti OR 'congenital heart malformation':ab,ti OR 'congenital heart malformations':ab,ti OR 'congenital heart abnormality':ab,ti OR 'congenital heart abnormalities':ab,ti OR 'congenital cardiac disease':ab,ti OR 'congenital cardiac diseases':ab,ti OR 'congenital cardiac defect':ab,ti OR 'congenital cardiac defects':ab,ti OR 'congenital cardiac malformation':ab,ti OR 'congenital cardiac malformations':ab,ti OR 'congenital cardiac abnormality':ab,ti OR 'congenital cardiac abnormalities':ab,ti OR 'congenital cardiovascular anomaly':ab,ti OR 'congenital cardiovascular anomalies':ab,ti OR 'congenital cardiovascular malformation':ab,ti OR 'congenital cardiovascular malformations':ab,ti OR 'congenital cardiovascular defect':ab,ti OR 'congenital cardiovascular defects':ab,ti OR 'aortic coarctation':ab,ti OR 'aortic coarctations':ab,ti OR 'coarctation of the aorta':ab,ti OR 'coarctation of aorta':ab,ti OR 'aorta coarctation':ab,ti OR 'aorta coarctations':ab,ti OR 'aorta dominant coarctation':ab,ti OR 'aorta dominant coarctations':ab,ti OR 'aortico-ventricular tunnel':ab,ti OR 'aortico-ventricular tunnels':ab,ti OR 'aortic right ventricular tunnel':ab,ti OR 'aortic right ventricular tunnels':ab,ti OR 'aortic left ventricular tunnel':ab,ti OR 'aortic left ventricular tunnels':ab,ti OR 'aortoventricular tunnel':ab,ti OR 'aortoventricular tunnels':ab,ti OR 'aorto ventricular tunnel':ab,ti OR 'aorto ventricular tunnels':ab,ti OR 'aorticoventricular tunnel':ab,ti OR 'aorticoventricular tunnels':ab,ti OR 'aortic ventricular tunnel':ab,ti OR 'aortic ventricular tunnels':ab,ti OR 'aortico left ventricular tunnel':ab,ti OR 'aortico left ventricular tunnels':ab,ti OR 'aorto left ventricular tunnel':ab,ti OR 'aorto left ventricular tunnels':ab,ti OR 'aortico right ventricular tunnel':ab,ti OR 'aortico right ventricular tunnels':ab,ti OR 'aorto right ventricular tunnel':ab,ti OR 'aorto right ventricular tunnels':ab,ti OR 'arrhythmogenic right ventricular dysplasia':ab,ti OR 'arrhythmogenic right ventricular cardiomyopathy dysplasia':ab,ti OR 'arvd-c':ab,ti OR 'arrhythmogenic right ventricular cardiomyopathy':ab,ti OR 'arrhythmogenic right ventricular dysplasia-cardiomyopathy':ab,ti OR 'bicuspid aortic valve disease':ab,ti OR 'aortic valve disease 1':ab,ti OR 'cor triatriatum':ab,ti OR 'subdivided left atrium':ab,ti OR 'subdivided left atriums':ab,ti OR 'triatrial heart':ab,ti OR 'triatrial hearts':ab,ti OR 'coronary vessel anomalies':ab,ti OR 'coronary vessel anomaly':ab,ti OR 'anomalous left coronary artery':ab,ti OR 'bland white garland syndrome':ab,ti OR alcapa:ab,ti OR 'myocardial bridging':ab,ti OR 'myocardial bridgings':ab,ti OR 'crisscross heart':ab,ti OR 'crisscross hearts':ab,ti OR 'criss cross heart':ab,ti OR 'criss cross hearts':ab,ti OR dextrocardia:ab,ti OR dextrocardias:ab,ti OR 'patent ductus arteriosus':ab,ti OR 'patency of the ductus arteriosus':ab,ti OR 'ebstein anomaly':ab,ti OR 'ebstein/s malformation':ab,ti OR 'ebstein malformation':ab,ti OR 'ebsteins malformation':ab,ti OR 'ebstein/s anomaly':ab,ti OR 'ebsteins anomaly':ab,ti OR 'ectopia cordis':ab,ti OR 'eisenmenger complex':ab,ti OR 'eisenmenger/s complex':ab,ti OR 'eisenmengers complex':ab,ti OR 'eisenmenger/s syndrome':ab,ti OR 'eisenmengers syndrome':ab,ti OR 'eisenmenger syndrome':ab,ti OR 'septal defect':ab,ti OR 'septal defects':ab,ti OR 'persistent truncus arteriosus':ab,ti OR 'endocardial cushion defects':ab,ti OR 'endocardial cushion defect':ab,ti OR 'persistent common atrioventricular canal':ab,ti OR 'persistent ostium primum':ab,ti OR 'patent oval foramen':ab,ti OR 'patent foramen ovale':ab,ti OR 'lutembacher syndrome':ab,ti OR 'lutembacher/s syndrome':ab,ti OR 'lutembachers syndrome':ab,ti OR 'double outlet right ventricle':ab,ti OR 'double-outlet right ventricles':ab,ti OR 'taussig bing anomaly':ab,ti OR 'heterotaxy syndrome':ab,ti OR 'heterotaxy syndromes':ab,ti OR 'visceral heterotaxy':ab,ti OR 'visceral heterotaxies':ab,ti OR 'situs ambiguus':ab,ti OR 'polysplenia syndrome':ab,ti OR 'polysplenia syndromes':ab,ti OR 'left atrial isomerism with polysplenia':ab,ti OR 'right atrial isomerism':ab,ti OR 'right atrial isomerisms':ab,ti OR 'asplenia syndrome':ab,ti OR 'asplenia syndromes':ab,ti OR 'right atrial isomerism with asplenia':ab,ti OR 'asplenia with cardiovascular anomalies':ab,ti OR 'ivemark syndrome':ab,ti OR 'left atrial isomerism':ab,ti OR 'left atrial isomerisms':ab,ti OR 'hypoplastic left heart syndrome':ab,ti OR 'left heart hypoplasia syndrome':ab,ti OR 'isolated noncompaction of the ventricular myocardium':ab,ti OR 'isolated non-compaction of the ventricular myocardium':ab,ti OR 'noncompaction of the left ventricular myocardium':ab,ti OR 'leopard syndrome':ab,ti OR 'lentiginosis cardiomyopathic':ab,ti OR 'lentiginosis cardiomyopathics':ab,ti OR 'progressive cardiomyopathic lentiginosis':ab,ti OR 'progressive cardiomyopathic lentiginoses':ab,ti OR 'cardio cutaneous syndrome':ab,ti OR 'cardiomyopathic lentiginosis':ab,ti OR 'cardiomyopathic lentiginoses':ab,ti OR levocardia:ab,ti OR 'quadricuspid aortic valve':ab,ti OR 'quadricuspid aortic valves':ab,ti OR 'quadricuspid aortic valvular disease':ab,ti OR 'tetralogy of fallot':ab,ti OR 'fallot tetralogy':ab,ti OR 'fallot/s tetralogy':ab,ti OR 'fallots tetralogy':ab,ti OR 'transposition of great vessels':ab,ti OR 'great vessels transposition':ab,ti OR 'great vessels transpositions':ab,ti OR 'transposition of the great arteries':ab,ti OR 'great arteries transposition':ab,ti OR 'great arteries transpositions':ab,ti OR 'congenitally corrected transposition':ab,ti OR 'congenitally corrected transpositions':ab,ti OR 'tricuspid atresia':ab,ti OR 'tricuspid atresias':ab,ti OR 'tricuspid valve atresias':ab,ti OR 'tricuspid valve atresia':ab,ti OR 'trilogy of fallot':ab,ti OR 'fallot trilogy':ab,ti OR 'fallot/s trilogy':ab,ti OR 'fallots trilogy':ab,ti OR 'univentricular heart':ab,ti OR 'univentricular hearts':ab,ti OR 'complex single ventricle':ab,ti OR 'complex single ventricles':ab,ti OR 'congenital heart block':ab,ti OR 'atrioventricular block':ab,ti OR 'congenital cardiac block':ab,ti OR 'congenital cardial block':ab,ti OR 'supravalvar aortic stenosis syndrome':ab,ti OR 'hypercalcemia supravalvar aortic stenosis':ab,ti OR 'pulmonary arterial hypertension':ab,ti OR 'pulmonary artery hypertension':ab,ti OR cardiology:ab,ti OR 'heart disease*':ab,ti OR 'heart disorder':ab,ti OR 'cardiac disease*':ab,ti OR 'cardiac disorder*':ab,ti OR arrhythmia*:ab,ti OR dysrhythmia*:ab,ti OR 'carcinoid heart disease*':ab,ti OR 'cardiac conduction':ab,ti OR 'high cardiac output':ab,ti OR 'low cardiac output':ab,ti OR 'cardiac tamponade*':ab,ti OR 'pericardial tamponade*':ab,ti OR cardiomegaly:ab,ti OR 'enlarged heart*':ab,ti OR 'cardiac hypertroph*':ab,ti OR 'heart hypertroph*':ab,ti OR cardiomyopath*:ab,ti OR myocardiopath*:ab,ti OR 'myocardial disease*':ab,ti OR cardiotoxicit*:ab,ti OR 'cardiac toxicit*':ab,ti OR endocarditis:ab,ti OR 'heart valve disease*':ab,ti OR 'valve disease*':ab,ti OR 'valve insufficiency':ab,ti OR 'valve stenosis':ab,ti OR 'valve prolapse':ab,ti OR 'valve atresia':ab,ti OR 'pulmonary atresia':ab,ti OR 'myocardial ischemia*':ab,ti OR 'ischemic heart disease*':ab,ti OR 'myocardial stunning':ab,ti OR 'myocardial hibernation':ab,ti OR 'stunned myocardium':ab,ti OR 'pericardial effusion*':ab,ti OR hemopericardium:ab,ti OR chylopericardium:ab,ti OR pericarditis:ab,ti OR pleuropericarditis:ab,ti OR pneumopericardium:ab,ti OR 'post-cardiac arrest syndrome*':ab,ti OR 'postresuscitation disease*':ab,ti OR 'postcardiac arrest syndrome*':ab,ti OR 'postpericardiotomy syndrome*':ab,ti OR 'postcommissurotomy syndrome*':ab,ti OR 'pulmonary heart disease*':ab,ti OR 'rheumatic heart disease*':ab,ti OR 'bouillaud disease':ab,ti OR 'bouillaud/s disease':ab,ti OR 'bouillauds disease':ab,ti OR 'ventricular dysfunction*':ab,ti OR 'ventricular outflow obstruction*':ab,ti OR 'sinus arrhythmia*':ab,ti OR 'sinoatrial arrhythmia*':ab,ti OR 'sick sinus syndrome':ab,ti OR 'sick sinus node syndrome':ab,ti OR 'sinus node dysfunction':ab,ti OR 'sinus node disease*':ab,ti OR 'cardiac sinus arrest*':ab,ti OR 'cardiac sinus pause*':ab,ti OR 'atrial fibrillation*':ab,ti OR 'auricular fibrillation*':ab,ti OR 'atrial flutter*':ab,ti OR 'auricular flutter*':ab,ti OR bradycardia*:ab,ti OR bradyarrhythmia*:ab,ti OR 'brugada syndrome':ab,ti OR 'sudden unexplained death syndrome':ab,ti OR 'sudden unexplained nocturnal death syndrome':ab,ti OR 'brugada ecg pattern':ab,ti OR 'brugada type ecg pattern':ab,ti OR 'premature beats*':ab,ti OR extrasystole:ab,ti OR 'premature cardiac complex*':ab,ti OR 'ectopic heartbeat*':ab,ti OR 'commotio cordis':ab,ti OR 'cardiac concussion*':ab,ti OR 'heart block*':ab,ti OR 'auriculo-ventricular dissociation*':ab,ti OR 'atrioventricular dissociation*':ab,ti OR 'a v dissociation*':ab,ti OR 'long qt syndrome':ab,ti OR parasystole*:ab,ti OR 'pre-excitation syndrome*':ab,ti OR 'preexcitation syndrome*':ab,ti OR tachycardia*:ab,ti OR tachyarrhythmia*:ab,ti OR 'ventricular fibrillation*':ab,ti OR 'ventricular flutter*':ab,ti OR 'dilated cardiomyopath*':ab,ti OR 'idiopathic cardiomyopath*':ab,ti OR 'congestive cardiomyopath*':ab,ti OR 'ventricular hypertroph*':ab,ti OR 'hypertrophic cardiomyopath*':ab,ti OR 'heart failure':ab,ti OR 'cardio-renal syndrome*':ab,ti OR 'reno-cardiac syndrome*':ab,ti OR 'renocardiac syndrome*':ab,ti OR 'cardiorenal syndrome*':ab,ti OR 'coronary disease*':ab,ti OR 'coronary aneurysm*':ab,ti OR 'coronary artery disease*':ab,ti OR 'left main disease*':ab,ti OR 'coronary arterioscleros*':ab,ti OR 'coronary atheroscleros*':ab,ti OR 'coronary occlusion*':ab,ti OR 'coronary stenos*':ab,ti OR 'coronary artery stenos*':ab,ti OR 'coronary restenos*':ab,ti OR 'coronary-subclavian steal syndrome*':ab,ti OR 'coronary thrombos*':ab,ti OR 'coronary vasospasm*':ab,ti OR 'coronary artery spasm*':ab,ti OR 'coronary spasm*':ab,ti OR 'myocardial infarct**':ab,ti OR 'cardiovascular stroke*':ab,ti OR 'heart attack*':ab,ti OR 'myocardial reperfusion injur*':ab,ti OR 'myocardial ischemic reperfusion injur*':ab,ti) AND (english:la OR spanish:la) NOT 'conference abstract':it AND [2000-2023]/py |
| CINAHL | (DE (Pediatrics OR child OR "Child, preschool” OR Infant OR adolescent "Infant, newborn”) OR TI (pediatric* OR newborn* OR neonat* OR infant* OR toddler* OR baby OR babies OR "school age*" OR "preschool age*" OR "pre school age" OR child* OR adolescen* OR teen* OR youth*) OR AB (pediatric* OR newborn* OR neonat* OR infant* OR toddler* OR baby OR babies OR "school age*" OR "preschool age*" OR "pre school age" OR child* OR adolescen* OR teen* OR youth*)) AND (DE ("palliative medicine” OR "palliative care nursing” OR “hospice nursing” OR hospices OR "hospice care" OR "palliative care" OR "terminal care”) OR TI ("palliative medicine" OR "supportive medicine" OR "palliative nursing" OR hospice* OR "bereavement care" OR "bereavement support" OR palliative OR "Supportive Care" OR "Supportive treatment*" OR "supportive therap*" OR "compassionate care" OR "terminal care" OR "end of life care") OR AB ("palliative medicine" OR "supportive medicine" OR "palliative nursing" OR hospice* OR "bereavement care" OR "bereavement support" OR palliative OR "Supportive Care" OR "Supportive treatment*" OR "supportive therap*" OR "compassionate care" OR "terminal care" OR "end of life care")) AND (DE ("Quality of Life" OR "Psychological Well-Being" OR "Personal Satisfaction" OR "patient satisfaction" OR "Job Satisfaction" OR pain OR "Stress, Psychological" OR "Outcome Assessment" OR "Treatment Outcomes" OR "Physician-Patient Relations" OR "Health Personnel" OR Physicians OR Nurses OR “health care delivery” OR "Intensive Care Units, Pediatric”) OR TI ("quality of life" OR "life quality" OR hrqol OR "wellbeing" OR "Well Being" OR "ill being" OR satisfaction OR satisfied OR "Job Satisfaction" OR "work satisfaction" OR pain OR suffer* OR stress* OR distress* OR outcome* OR "Physician-Patient Relation*" OR "Doctor Patient Relation*" OR "health personnel" OR "medical personnel" OR "Health Care Provider*" OR "Healthcare Provider*" OR "medical staff" OR "health care staff" OR "healthcare staff" OR "Health Care Professional*" OR "Healthcare Professional*" OR "medical professional*" OR physician* OR doctor* OR nurse* OR "Delivery of Health Care" OR "Delivery of Healthcare" OR "Health Care Delivery" OR "Healthcare Delivery" OR "Health Care System*" OR "Healthcare System*" OR "Pediatric Intensive Care Unit*" OR PICU OR "cardiac intensive care unit*" OR CICU OR) OR AB ("quality of life" OR "life quality" OR hrqol OR "wellbeing" OR "Well Being" OR "ill being" OR satisfaction OR satisfied OR "Job Satisfaction" OR "work satisfaction" OR pain OR suffer* OR stress* OR distress* OR outcome* OR "Physician-Patient Relation*" OR "Doctor Patient Relation*" OR "health personnel" OR "medical personnel" OR "Health Care Provider*" OR "Healthcare Provider*" OR "medical staff" OR "health care staff" OR "healthcare staff" OR "Health Care Professional*" OR "Healthcare Professional*" OR "medical professional*" OR physician* OR doctor* OR nurse* OR "Delivery of Health Care" OR "Delivery of Healthcare" OR "Health Care Delivery" OR "Healthcare Delivery" OR "Health Care System*" OR "Healthcare System*" OR "Pediatric Intensive Care Unit*" OR PICU OR "cardiac intensive care unit*" OR CICU)) AND (DE ("Heart Defects, Congenital" OR "Aortic Coarctation" OR "Aortico-Ventricular Tunnel" OR "Arrhythmogenic Right Ventricular Dysplasia" OR "Bicuspid Aortic Valve Disease" OR "Aortic Valve Disease 1" OR "Cor Triatriatum" OR "Coronary Vessel Anomalies" OR "Anomalous Left Coronary Artery" OR "Bland White Garland Syndrome" OR "Myocardial Bridging" OR "Crisscross Heart" OR Dextrocardia OR "Ductus Arteriosus, Patent" OR "Ebstein Anomaly" OR "Ectopia Cordis" OR "Eisenmenger Complex" OR "heart septal defects" OR "Endocardial Cushion Defects" OR "Truncus Arteriosus, Persistent" OR "Heart Septal Defects, Atrial" OR "Foramen Ovale, Patent" OR "Lutembacher Syndrome" OR "Double Outlet Right Ventricle" OR "Heterotaxy Syndrome" OR "Hypoplastic Left Heart Syndrome" "Isolated Noncompaction of the Ventricular Myocardium" OR "LEOPARD Syndrome" OR Levocardia OR "Quadricuspid Aortic Valve" OR "Tetralogy of Fallot" OR "Transposition of Great Vessels" OR "Congenitally Corrected Transposition of the Great Arteries" OR "Tricuspid Atresia" OR "Trilogy of Fallot" OR "Univentricular Heart" OR "Atrioventricular Block" OR "Pulmonary Arterial Hypertension" OR Cardiology OR "Heart Diseases" OR "Arrhythmias, Cardiac" OR "Carcinoid Heart Disease" OR "Cardiac Output, High" OR "Cardiac Conduction System Disease" OR "Cardiac Output, Low" OR "Cardiac Tamponade" OR Cardiomegaly OR Cardiomyopathies OR Endocarditis OR Cardiotoxicity OR "Heart Valve Diseases" OR "pulmonary atresia" OR "Myocardial Ischemia" OR "Myocardial Stunning" OR "Pericardial Effusion" OR Pericarditis OR Pneumopericardium OR "Post-Cardiac Arrest Syndrome" OR "Postpericardiotomy Syndrome" OR "Pulmonary Heart Disease" OR "Rheumatic Heart Disease" OR "Ventricular Dysfunction" OR "Ventricular Outflow Obstruction" *" OR "Arrhythmia, Sinus" OR "Sick Sinus Syndrome" OR "Sinus Arrest, Cardiac" OR "Atrial Fibrillation" OR "Atrial Flutter" OR "Brugada Syndrome" OR Bradycardia OR "Cardiac Complexes, Premature" OR "Heart Block" OR "Commotio Cordis" OR "Long QT Syndrome" OR Parasystole OR "Pre-Excitation Syndromes" OR "Ventricular Fibrillation" OR Tachycardia OR "Ventricular Flutter" OR "Cardiomyopathy, Dilated" OR "Hypertrophy, Left Ventricular" OR "Hypertrophy, Right Ventricular" OR "Heart Failure" OR "Cardio-Renal Syndrome" OR "Coronary Disease" OR "Coronary Aneurysm" OR "Coronary Artery Disease" OR "Coronary Occlusion" OR "Coronary Stenosis" OR "Coronary Restenosis" OR "Coronary-Subclavian Steal Syndrome" OR "Coronary Thrombosis" OR "Coronary Vasospasm" OR "Myocardial Infarction" OR "Myocardial Reperfusion Injury") OR TI ("congenital heart disease*" OR CHD OR "congenital heart defect*" OR "congenital heart malformation*" OR "congenital heart abnormalit*" OR "congenital cardiac disease*" OR "congenital cardiac defect*" OR "congenital cardiac malformation*" OR "congenital cardiac abnormalit*" OR "congenital cardiovascular anomal*" OR "congenital cardiovascular malformation*" OR "congenital cardiovascular defect*" OR "Aortic Coarctation*" OR "Coarctation of the Aorta" OR "Coarctation of Aorta" OR "Aorta Coarctation*" OR "Aorta Dominant Coarctation" OR "Aortico-Ventricular Tunnel*" OR "Aortic Right Ventricular Tunnel*" OR "Aortic Left Ventricular Tunnel*" OR "Aortoventricular Tunnel*" OR "Aorto Ventricular Tunnel*" OR "Aorticoventricular Tunnel*" OR "Aortic Ventricular Tunnel*" OR "Aortico Left Ventricular Tunnel*" OR "Aorto Left Ventricular Tunnel*" OR "Aortico Right Ventricular Tunnel*" OR "Aorto Right Ventricular Tunnel*" OR "Arrhythmogenic Right Ventricular Dysplasia" OR "Arrhythmogenic Right Ventricular Cardiomyopathy Dysplasia" OR "ARVD-C" OR "Arrhythmogenic Right Ventricular Cardiomyopathy" OR "Arrhythmogenic Right Ventricular Dysplasia-Cardiomyopathy" OR "Bicuspid Aortic Valve Disease*" OR "Cor Triatriatum" OR "Subdivided Left Atrium*" OR "Triatrial Heart*" OR "Coronary Vessel Anomal*" OR "Anomalous Left Coronary Artery" OR "Bland White Garland Syndrome" OR ALCAPA OR "Myocardial Bridging*" OR "Myocardial Bridgings" OR "Crisscross Heart*" OR "Criss cross Heart*" OR Dextrocardia* OR "Patent Ductus Arteriosus" OR "Patency of the Ductus Arteriosus" OR "Ebstein Anomaly" OR "Ebstein's Malformation" OR "Ebstein Malformation" OR "Ebsteins Malformation" OR "Ebstein's Anomaly" OR "Ebsteins Anomaly" OR "Ectopia Cordis" OR "Eisenmenger Complex" OR "Eisenmenger's Complex" OR "Eisenmengers Complex" OR "Eisenmenger's Syndrome" OR "Eisenmengers Syndrome" OR "Eisenmenger Syndrome" OR "septal defect*" OR "Persistent Truncus Arteriosus" OR "Endocardial Cushion Defect*" OR "Persistent Common Atrioventricular Canal" OR "Persistent Ostium Primum" OR "Patent Oval Foramen" OR "Patent Foramen Ovale" OR "Lutembacher Syndrome" OR "Lutembacher's Syndrome" OR "Lutembachers Syndrome" OR "Double Outlet Right Ventricle*" OR "Taussig Bing Anomaly" OR "Heterotaxy Syndrome*" OR "Visceral Heterotax*" OR "Situs Ambiguus" OR "Polysplenia Syndrome*" OR "Left Atrial Isomerism with Polysplenia" OR "Right Atrial Isomerism*" OR "Asplenia Syndrome*" OR "Right Atrial Isomerism with Asplenia" OR "Asplenia with Cardiovascular Anomalies" OR "Ivemark Syndrome" OR "Left Atrial Isomerism*" OR "Hypoplastic Left Heart Syndrome" OR "Left Heart Hypoplasia Syndrome" OR "Isolated Noncompaction of the Ventricular Myocardium" OR "Isolated Non-compaction of the Ventricular Myocardium" OR "Noncompaction of the Left Ventricular Myocardium" OR "LEOPARD Syndrome" OR "Lentiginosis Cardiomyopathic" OR "Progressive Cardiomyopathic Lentiginos*" OR "Cardio Cutaneous Syndrome" OR "Cardiomyopathic Lentiginos*" OR Levocardia OR OR "Quadricuspid Aortic Valve*" OR "Quadricuspid Aortic Valvular Disease" OR "Tetralogy of Fallot" OR "Fallot Tetralogy" OR "Fallot's Tetralogy" OR "Fallots Tetralogy" OR "Transposition of Great Vessels" OR "Great Vessels Transposition*" OR "Transposition of the Great Arteries" OR "Great Arteries Transposition*" OR "Congenitally Corrected Transposition*" OR "Tricuspid Atresia*" OR "Tricuspid Valve Atresia*" OR "Trilogy of Fallot" OR "Fallot Trilogy" OR "Fallot's Trilogy" OR "Fallots Trilogy" OR "Univentricular Heart*" OR "Complex Single Ventricle*" OR "congenital heart block" OR "Atrioventricular Block" OR "congenital cardiac block" OR "congenital cardial block" OR "Supravalvar Aortic Stenosis Syndrome" OR "Hypercalcemia Supravalvar Aortic Stenosis" OR "Pulmonary Arterial Hypertension" OR "Pulmonary Artery Hypertension" OR cardiology OR "Heart Disease*" OR "heart disorder" OR "cardiac disease*" OR "cardiac disorder*" OR arrhythmia* OR dysrhythmia* OR "Carcinoid Heart Disease*" OR "cardiac conduction" OR "high cardiac output" OR "low cardiac output" OR "Cardiac Tamponade*" OR "Pericardial Tamponade*" OR Cardiomegaly OR "Enlarged Heart*" OR "Cardiac Hypertroph*" OR "Heart Hypertroph*" OR Cardiomyopath* OR Myocardiopath* OR "Myocardial Disease*" OR Cardiotoxicit* OR "Cardiac Toxicit*" OR Endocarditis OR "Heart Valve Disease*" OR "valve disease*" OR "valve insufficiency" OR "valve stenosis" OR "valve prolapse" OR "valve atresia" OR "pulmonary atresia" OR "Myocardial Ischemia*" OR "Ischemic Heart Disease*" OR "Myocardial Stunning" OR "Myocardial Hibernation" OR "Stunned Myocardium" OR "Pericardial Effusion*" OR Hemopericardium OR Chylopericardium OR Pericarditis OR Pleuropericarditis OR Pneumopericardium OR "Post-Cardiac Arrest Syndrome*" OR "Postresuscitation Disease*" OR "Postcardiac Arrest Syndrome*" OR "Postpericardiotomy Syndrome*" OR "Postcommissurotomy Syndrome*" OR "Pulmonary Heart Disease*" OR "Rheumatic Heart Disease*" OR "Bouillaud Disease" OR "Bouillaud's Disease" OR "Bouillauds Disease" OR "Ventricular Dysfunction*" OR "Ventricular Outflow Obstruction” OR "Sinus Arrhythmia*" OR "Sinoatrial Arrhythmia*" OR "Sick Sinus Syndrome" OR "Sick Sinus Node Syndrome" OR "Sinus Node Dysfunction" OR "Sinus Node Disease*" OR "Cardiac Sinus Arrest*" OR "Cardiac Sinus Pause*" OR "Atrial Fibrillation*" OR "Auricular Fibrillation*" OR "Atrial Flutter*" OR "Auricular Flutter*" OR Bradycardia* OR Bradyarrhythmia* OR "Brugada Syndrome" OR "Sudden Unexplained Death Syndrome" OR "Sudden Unexplained Nocturnal Death Syndrome" OR "Brugada ECG Pattern" OR "Brugada Type ECG Pattern" OR "Premature Beats*" OR Extrasystole OR "Premature Cardiac Complex" OR "Ectopic Heartbeat*" OR "Commotio Cordis" OR "Cardiac Concussion*" OR "Heart Block*" OR "Auriculo-Ventricular Dissociation*" OR "Atrioventricular Dissociation*" OR "A V Dissociation*" OR "Long QT Syndrome" OR Parasystole* OR "Pre-Excitation Syndrome*" OR "Preexcitation Syndrome*" OR Tachycardia* OR Tachyarrhythmia* OR "Ventricular Fibrillation*" OR "Ventricular Flutter*" OR "Dilated Cardiomyopath*" OR "Idiopathic Cardiomyopath*" OR "Congestive Cardiomyopath*" OR "ventricular hypertroph*" OR "Hypertrophic Cardiomyopath*" OR "Heart Failure" OR "Cardio-Renal Syndrome*" OR "Reno-Cardiac Syndrome*" OR "Renocardiac Syndrome*" OR "Cardiorenal Syndrome*" OR "Coronary Disease*" OR "Coronary Aneurysm*" OR "Coronary Artery Disease*" OR "Left Main Disease*" OR "Coronary Arterioscleros*" OR "Coronary Atheroscleros*" OR "Coronary Occlusion*" OR "Coronary Stenos*" OR "Coronary Artery Stenos*" OR "Coronary Restenos*" OR "Coronary-Subclavian Steal Syndrome*" OR "Coronary Thrombos*" OR "Coronary Vasospasm*" OR "Coronary Artery Spasm*" OR "Coronary Spasm*" OR "Myocardial Infarct**" OR "Cardiovascular Stroke*" OR "Heart Attack*" OR "Myocardial Reperfusion Injur*" OR "Myocardial Ischemic Reperfusion Injur*") OR AB ("congenital heart disease*" OR CHD OR "congenital heart defect*" OR "congenital heart malformation*" OR "congenital heart abnormalit*" OR "congenital cardiac disease*" OR "congenital cardiac defect*" OR "congenital cardiac malformation*" OR "congenital cardiac abnormalit*" OR "congenital cardiovascular anomal*" OR "congenital cardiovascular malformation*" OR "congenital cardiovascular defect*" OR "Aortic Coarctation*" OR "Coarctation of the Aorta" OR "Coarctation of Aorta" OR "Aorta Coarctation*" OR "Aorta Dominant Coarctation" OR "Aortico-Ventricular Tunnel*" OR "Aortic Right Ventricular Tunnel*" OR "Aortic Left Ventricular Tunnel*" OR "Aortoventricular Tunnel*" OR "Aorto Ventricular Tunnel*" OR "Aorticoventricular Tunnel*" OR "Aortic Ventricular Tunnel*" OR "Aortico Left Ventricular Tunnel*" OR "Aorto Left Ventricular Tunnel*" OR "Aortico Right Ventricular Tunnel*" OR "Aorto Right Ventricular Tunnel*" OR "Arrhythmogenic Right Ventricular Dysplasia" OR "Arrhythmogenic Right Ventricular Cardiomyopathy Dysplasia" OR "ARVD-C" OR "Arrhythmogenic Right Ventricular Cardiomyopathy" OR "Arrhythmogenic Right Ventricular Dysplasia-Cardiomyopathy" OR "Bicuspid Aortic Valve Disease*" OR "Cor Triatriatum" OR "Subdivided Left Atrium*" OR "Triatrial Heart*" OR "Coronary Vessel Anomal*" OR "Anomalous Left Coronary Artery" OR "Bland White Garland Syndrome" OR ALCAPA OR "Myocardial Bridging*" OR "Myocardial Bridgings" OR "Crisscross Heart*" OR "Criss cross Heart*" OR Dextrocardia* OR "Patent Ductus Arteriosus" OR "Patency of the Ductus Arteriosus" OR "Ebstein Anomaly" OR "Ebstein's Malformation" OR "Ebstein Malformation" OR "Ebsteins Malformation" OR "Ebstein's Anomaly" OR "Ebsteins Anomaly" OR "Ectopia Cordis" OR "Eisenmenger Complex" OR "Eisenmenger's Complex" OR "Eisenmengers Complex" OR "Eisenmenger's Syndrome" OR "Eisenmengers Syndrome" OR "Eisenmenger Syndrome" OR "septal defect*" OR "Persistent Truncus Arteriosus" OR "Endocardial Cushion Defect*" OR "Persistent Common Atrioventricular Canal" OR "Persistent Ostium Primum" OR "Patent Oval Foramen" OR "Patent Foramen Ovale" OR "Lutembacher Syndrome" OR "Lutembacher's Syndrome" OR "Lutembachers Syndrome" OR "Double Outlet Right Ventricle*" OR "Taussig Bing Anomaly" OR "Heterotaxy Syndrome*" OR "Visceral Heterotax*" OR "Situs Ambiguus" OR "Polysplenia Syndrome*" OR "Left Atrial Isomerism with Polysplenia" OR "Right Atrial Isomerism*" OR "Asplenia Syndrome*" OR "Right Atrial Isomerism with Asplenia" OR "Asplenia with Cardiovascular Anomalies" OR "Ivemark Syndrome" OR "Left Atrial Isomerism*" OR "Hypoplastic Left Heart Syndrome" OR "Left Heart Hypoplasia Syndrome" OR "Isolated Noncompaction of the Ventricular Myocardium" OR "Isolated Non-compaction of the Ventricular Myocardium" OR "Noncompaction of the Left Ventricular Myocardium" OR "LEOPARD Syndrome" OR "Lentiginosis Cardiomyopathic" OR "Progressive Cardiomyopathic Lentiginos*" OR "Cardio Cutaneous Syndrome" OR "Cardiomyopathic Lentiginos*" OR Levocardia OR "Quadricuspid Aortic Valve*" OR "Quadricuspid Aortic Valvular Disease" OR "Tetralogy of Fallot" OR "Fallot Tetralogy" OR "Fallot's Tetralogy" OR "Fallots Tetralogy" OR "Transposition of Great Vessels" OR "Great Vessels Transposition*" OR "Transposition of the Great Arteries" OR "Great Arteries Transposition*" OR "Congenitally Corrected Transposition*" OR "Tricuspid Atresia*" OR "Tricuspid Valve Atresia*" OR "Trilogy of Fallot" OR "Fallot Trilogy" OR "Fallot's Trilogy" OR "Fallots Trilogy" OR "Univentricular Heart*" OR "Complex Single Ventricle*" OR "congenital heart block" OR "Atrioventricular Block" OR "congenital cardiac block" OR "congenital cardial block" OR "Supravalvar Aortic Stenosis Syndrome" OR "Hypercalcemia Supravalvar Aortic Stenosis" OR "Pulmonary Arterial Hypertension" OR "Pulmonary Artery Hypertension" OR cardiology OR "Heart Disease*" OR "heart disorder" OR "cardiac disease*" OR "cardiac disorder*" OR arrhythmia* OR dysrhythmia* OR "Carcinoid Heart Disease*" OR "cardiac conduction" OR "high cardiac output" OR "low cardiac output" OR "Cardiac Tamponade*" OR "Pericardial Tamponade*" OR Cardiomegaly OR "Enlarged Heart*" OR "Cardiac Hypertroph*" OR "Heart Hypertroph*" OR Cardiomyopath* OR Myocardiopath* OR "Myocardial Disease*" OR Cardiotoxicit* OR "Cardiac Toxicit*" OR Endocarditis OR "Heart Valve Disease*" OR "valve disease*" OR "valve insufficiency" OR "valve stenosis" OR "valve prolapse" OR "valve atresia" OR "pulmonary atresia" OR "Myocardial Ischemia*" OR "Ischemic Heart Disease*" OR "Myocardial Stunning" OR "Myocardial Hibernation" OR "Stunned Myocardium" OR "Pericardial Effusion*" OR Hemopericardium OR Chylopericardium OR Pericarditis OR Pleuropericarditis OR Pneumopericardium OR "Post-Cardiac Arrest Syndrome*" OR "Postresuscitation Disease*" OR "Postcardiac Arrest Syndrome*" OR "Postpericardiotomy Syndrome*" OR "Postcommissurotomy Syndrome*" OR "Pulmonary Heart Disease*" OR "Rheumatic Heart Disease*" OR "Bouillaud Disease" OR "Bouillaud's Disease" OR "Bouillauds Disease" OR "Ventricular Dysfunction*" OR "Ventricular Outflow Obstruction” OR "Sinus Arrhythmia*" OR "Sinoatrial Arrhythmia*" OR "Sick Sinus Syndrome" OR "Sick Sinus Node Syndrome" OR "Sinus Node Dysfunction" OR "Sinus Node Disease*" OR "Cardiac Sinus Arrest*" OR "Cardiac Sinus Pause*" OR "Atrial Fibrillation*" OR "Auricular Fibrillation*" OR "Atrial Flutter*" OR "Auricular Flutter*" OR Bradycardia* OR Bradyarrhythmia* OR "Brugada Syndrome" OR "Sudden Unexplained Death Syndrome" OR "Sudden Unexplained Nocturnal Death Syndrome" OR "Brugada ECG Pattern" OR "Brugada Type ECG Pattern" OR "Premature Beats*" OR Extrasystole OR "Premature Cardiac Complex" OR "Ectopic Heartbeat*" OR "Commotio Cordis" OR "Cardiac Concussion*" OR "Heart Block*" OR "Auriculo-Ventricular Dissociation*" OR "Atrioventricular Dissociation*" OR "A V Dissociation*" OR "Long QT Syndrome" OR Parasystole* OR "Pre-Excitation Syndrome*" OR "Preexcitation Syndrome*" OR Tachycardia* OR Tachyarrhythmia* OR "Ventricular Fibrillation*" OR "Ventricular Flutter*" OR "Dilated Cardiomyopath*" OR "Idiopathic Cardiomyopath*" OR "Congestive Cardiomyopath*" OR "ventricular hypertroph*" OR "Hypertrophic Cardiomyopath*" OR "Heart Failure" OR "Cardio-Renal Syndrome*" OR "Reno-Cardiac Syndrome*" OR "Renocardiac Syndrome*" OR "Cardiorenal Syndrome*" OR "Coronary Disease*" OR "Coronary Aneurysm*" OR "Coronary Artery Disease*" OR "Left Main Disease*" OR "Coronary Arterioscleros*" OR "Coronary Atheroscleros*" OR "Coronary Occlusion*" OR "Coronary Stenos*" OR "Coronary Artery Stenos*" OR "Coronary Restenos*" OR "Coronary-Subclavian Steal Syndrome*" OR "Coronary Thrombos*" OR "Coronary Vasospasm*" OR "Coronary Artery Spasm*" OR "Coronary Spasm*" OR "Myocardial Infarct**" OR "Cardiovascular Stroke*" OR "Heart Attack*" OR "Myocardial Reperfusion Injur*" OR "Myocardial Ischemic Reperfusion Injur*")) AND (LA (english OR spanish) AND (PY 2000-2023) |
| Web of Science | TS=(pediatric* OR newborn* OR neonat* OR infant* OR toddler* OR baby OR babies OR "school age*" OR "preschool age*" OR "pre school age" OR child* OR adolescen* OR teen* OR youth*) AND TS=("palliative medicine" OR "supportive medicine" OR "palliative nursing" OR hospice* OR "bereavement care" OR "bereavement support" OR palliative OR "Supportive Care" OR "Supportive treatment*" OR "supportive therap*" OR "compassionate care" OR "terminal care" OR "end of life care") AND TS= ("quality of life" OR "life quality" OR hrqol OR "wellbeing" OR "Well Being" OR "ill being" OR satisfaction OR satisfied OR "Job Satisfaction" OR "work satisfaction" OR pain OR suffer* OR stress* OR distress* OR outcome* OR "Physician-Patient Relation*" OR "Doctor Patient Relation*" OR "health personnel" OR "medical personnel" OR "Health Care Provider*" OR "Healthcare Provider*" OR "medical staff" OR "health care staff" OR "healthcare staff" OR "Health Care Professional*" OR "Healthcare Professional*" OR "medical professional*" OR physician* OR doctor* OR nurse* OR "Delivery of Health Care" OR "Delivery of Healthcare" OR "Health Care Delivery" OR "Healthcare Delivery" OR "Health Care System*" OR "Healthcare System*" OR "Pediatric Intensive Care Unit*" OR PICU OR "cardiac intensive care unit*" OR CICU) AND TS= ("congenital heart disease*" OR CHD OR "congenital heart defect*" OR "congenital heart malformation*" OR "congenital heart abnormalit*" OR "congenital cardiac disease*" OR "congenital cardiac defect*" OR "congenital cardiac malformation*" OR "congenital cardiac abnormalit*" OR "congenital cardiovascular anomal*" OR "congenital cardiovascular malformation*" OR "congenital cardiovascular defect*" OR "Aortic Coarctation*" OR "Coarctation of the Aorta" OR "Coarctation of Aorta" OR "Aorta Coarctation*" OR "Aorta Dominant Coarctation" OR "Aortico-Ventricular Tunnel*" OR "Aortic Right Ventricular Tunnel*" OR "Aortic Left Ventricular Tunnel*" OR "Aortoventricular Tunnel*" OR "Aorto Ventricular Tunnel*" OR "Aorticoventricular Tunnel*" OR "Aortic Ventricular Tunnel*" OR "Aortico Left Ventricular Tunnel*" OR "Aorto Left Ventricular Tunnel*" OR "Aortico Right Ventricular Tunnel*" OR "Aorto Right Ventricular Tunnel*" OR "Arrhythmogenic Right Ventricular Dysplasia" OR "Arrhythmogenic Right Ventricular Cardiomyopathy Dysplasia" OR "ARVD-C" OR "Arrhythmogenic Right Ventricular Cardiomyopathy" OR "Arrhythmogenic Right Ventricular Dysplasia-Cardiomyopathy" OR "Bicuspid Aortic Valve Disease*" OR "Cor Triatriatum" OR "Subdivided Left Atrium*" OR "Triatrial Heart*" OR "Coronary Vessel Anomal*" OR "Anomalous Left Coronary Artery" OR "Bland White Garland Syndrome" OR ALCAPA OR "Myocardial Bridging*" OR "Myocardial Bridgings" OR "Crisscross Heart*" OR "Criss cross Heart*" OR Dextrocardia* OR "Patent Ductus Arteriosus" OR "Patency of the Ductus Arteriosus" OR "Ebstein Anomaly" OR "Ebstein's Malformation" OR "Ebstein Malformation" OR "Ebsteins Malformation" OR "Ebstein's Anomaly" OR "Ebsteins Anomaly" OR "Ectopia Cordis" OR "Eisenmenger Complex" OR "Eisenmenger's Complex" OR "Eisenmengers Complex" OR "Eisenmenger's Syndrome" OR "Eisenmengers Syndrome" OR "Eisenmenger Syndrome" OR "septal defect*" OR "Persistent Truncus Arteriosus" OR "Endocardial Cushion Defect*" OR "Persistent Common Atrioventricular Canal" OR "Persistent Ostium Primum" OR "Patent Oval Foramen" OR "Patent Foramen Ovale" OR "Lutembacher Syndrome" OR "Lutembacher's Syndrome" OR "Lutembachers Syndrome" OR "Double Outlet Right Ventricle*" OR "Taussig Bing Anomaly" OR "Heterotaxy Syndrome*" OR "Visceral Heterotax*" OR "Situs Ambiguus" OR "Polysplenia Syndrome*" OR "Left Atrial Isomerism with Polysplenia" OR "Right Atrial Isomerism*" OR "Asplenia Syndrome*" OR "Right Atrial Isomerism with Asplenia" OR "Asplenia with Cardiovascular Anomalies" OR "Ivemark Syndrome" OR "Left Atrial Isomerism*" OR "Hypoplastic Left Heart Syndrome" OR "Left Heart Hypoplasia Syndrome" OR "Isolated Noncompaction of the Ventricular Myocardium" OR "Isolated Non-compaction of the Ventricular Myocardium" OR "Noncompaction of the Left Ventricular Myocardium" OR "LEOPARD Syndrome" OR "Lentiginosis Cardiomyopathic" OR "Progressive Cardiomyopathic Lentiginos*" OR "Cardio Cutaneous Syndrome" OR "Cardiomyopathic Lentiginos*" OR Levocardia OR "Quadricuspid Aortic Valve*" OR "Quadricuspid Aortic Valvular Disease" OR "Tetralogy of Fallot" OR "Fallot Tetralogy" OR "Fallot's Tetralogy" OR "Fallots Tetralogy" OR "Transposition of Great Vessels" OR "Great Vessels Transposition*" OR "Transposition of the Great Arteries" OR "Great Arteries Transposition*" OR "Congenitally Corrected Transposition*" OR "Tricuspid Atresia*" OR "Tricuspid Valve Atresia*" OR "Trilogy of Fallot" OR "Fallot Trilogy" OR "Fallot's Trilogy" OR "Fallots Trilogy" OR "Univentricular Heart*" OR "Complex Single Ventricle*" OR "congenital heart block" OR "Atrioventricular Block" OR "congenital cardiac block" OR "congenital cardial block" OR "Supravalvar Aortic Stenosis Syndrome" OR "Hypercalcemia Supravalvar Aortic Stenosis" OR "Pulmonary Arterial Hypertension" OR "Pulmonary Artery Hypertension" OR cardiology OR "Heart Disease*" OR "heart disorder" OR "cardiac disease*" OR "cardiac disorder*" OR arrhythmia* OR dysrhythmia* OR "Carcinoid Heart Disease*" OR "cardiac conduction" OR "high cardiac output" OR "low cardiac output" OR "Cardiac Tamponade*" OR "Pericardial Tamponade*" OR Cardiomegaly OR "Enlarged Heart*" OR "Cardiac Hypertroph*" OR "Heart Hypertroph*" OR Cardiomyopath* OR Myocardiopath* OR "Myocardial Disease*" OR Cardiotoxicit* OR "Cardiac Toxicit*" OR Endocarditis OR "Heart Valve Disease*" OR "valve disease*" OR "valve insufficiency" OR "valve stenosis" OR "valve prolapse" OR "valve atresia" OR "pulmonary atresia" OR "Myocardial Ischemia*" OR "Ischemic Heart Disease*" OR "Myocardial Stunning" OR "Myocardial Hibernation" OR "Stunned Myocardium" OR "Pericardial Effusion*" OR Hemopericardium OR Chylopericardium OR Pericarditis OR Pleuropericarditis OR Pneumopericardium OR "Post-Cardiac Arrest Syndrome*" OR "Postresuscitation Disease*" OR "Postcardiac Arrest Syndrome*" OR "Postpericardiotomy Syndrome*" OR "Postcommissurotomy Syndrome*" OR "Pulmonary Heart Disease*" OR "Rheumatic Heart Disease*" OR "Bouillaud Disease" OR "Bouillaud's Disease" OR "Bouillauds Disease" OR "Ventricular Dysfunction*" OR "Ventricular Outflow Obstruction” OR "Sinus Arrhythmia*" OR "Sinoatrial Arrhythmia*" OR "Sick Sinus Syndrome" OR "Sick Sinus Node Syndrome" OR "Sinus Node Dysfunction" OR "Sinus Node Disease*" OR "Cardiac Sinus Arrest*" OR "Cardiac Sinus Pause*" OR "Atrial Fibrillation*" OR "Auricular Fibrillation*" OR "Atrial Flutter*" OR "Auricular Flutter*" OR Bradycardia* OR Bradyarrhythmia* OR "Brugada Syndrome" OR "Sudden Unexplained Death Syndrome" OR "Sudden Unexplained Nocturnal Death Syndrome" OR "Brugada ECG Pattern" OR "Brugada Type ECG Pattern" OR "Premature Beats*" OR Extrasystole OR "Premature Cardiac Complex" OR "Ectopic Heartbeat*" OR "Commotio Cordis" OR "Cardiac Concussion*" OR "Heart Block*" OR "Auriculo-Ventricular Dissociation*" OR "Atrioventricular Dissociation*" OR "A V Dissociation*" OR "Long QT Syndrome" OR Parasystole* OR "Pre-Excitation Syndrome*" OR "Preexcitation Syndrome*" OR Tachycardia* OR Tachyarrhythmia* OR "Ventricular Fibrillation*" OR "Ventricular Flutter*" OR "Dilated Cardiomyopath*" OR "Idiopathic Cardiomyopath*" OR "Congestive Cardiomyopath*" OR "ventricular hypertroph*" OR "Hypertrophic Cardiomyopath*" OR "Heart Failure" OR "Cardio-Renal Syndrome*" OR "Reno-Cardiac Syndrome*" OR "Renocardiac Syndrome*" OR "Cardiorenal Syndrome*" OR "Coronary Disease*" OR "Coronary Aneurysm*" OR "Coronary Artery Disease*" OR "Left Main Disease*" OR "Coronary Arterioscleros*" OR "Coronary Atheroscleros*" OR "Coronary Occlusion*" OR "Coronary Stenos*" OR "Coronary Artery Stenos*" OR "Coronary Restenos*" OR "Coronary-Subclavian Steal Syndrome*" OR "Coronary Thrombos*" OR "Coronary Vasospasm*" OR "Coronary Artery Spasm*" OR "Coronary Spasm*" OR "Myocardial Infarct**" OR "Cardiovascular Stroke*" OR "Heart Attack*" OR "Myocardial Reperfusion Injur*" OR "Myocardial Ischemic Reperfusion Injur*") AND LA=(english or Spanish) |
| CENTRAL | ID Search Hits  #1 MeSH descriptor: [Pediatrics] explode all trees 1043  #2 MeSH descriptor: [Infant, Newborn] explode all trees 23977  #3 MeSH descriptor: [Infant] explode all trees 45994  #4 MeSH descriptor: [Child, Preschool] explode all trees 39429  #5 MeSH descriptor: [Child] explode all trees 81699  #6 MeSH descriptor: [Adolescent] explode all trees 136681  #7 pediatric* OR newborn* OR neonat* OR infant* OR toddler* OR baby OR babies OR (school NEXT age*) OR (preschool NEXT age) OR (pre NEXT school NEXT age) OR child* OR adolescen* OR teen* OR youth* 388816  #8 #1 OR #2 OR #3 OR #4 OR #5 OR #6 OR #7 388830  #9 MeSH descriptor: [Palliative Medicine] explode all trees 4  #10 MeSH descriptor: [Hospice and Palliative Care Nursing] explode all trees 98  #11 MeSH descriptor: [Hospices] explode all trees 78  #12 MeSH descriptor: [Hospice Care] explode all trees 178  #13 MeSH descriptor: [Palliative Care] explode all trees 2597  #14 MeSH descriptor: [Terminal Care] explode all trees 753  #15 (palliative NEXT medicine) OR (supportive NEXT medicine) OR (palliative NEXT nursing) OR hospice* OR (bereavement NEXT care) OR (bereavement NEXT support) OR palliative OR (Supportive NEXT Care) OR (Supportive NEXT treatment*) OR (supportive NEXT therap*) OR (compassionate NEXT care) OR (terminal NEXT care) OR (end NEXT of NEXT life NEXT care) 20968  #16 #9 OR #10 OR #11 OR #12 OR #13 OR #14 OR #15 21013  #17 MeSH descriptor: [Quality of Life] explode all trees 43646  #18 MeSH descriptor: [Psychological Well-Being] explode all trees 35  #19 MeSH descriptor: [Personal Satisfaction] explode all trees 1516  #20 MeSH descriptor: [Patient Satisfaction] explode all trees 15451  #21 MeSH descriptor: [Job Satisfaction] explode all trees 391  #22 MeSH descriptor: [Pain] explode all trees 71941  #23 MeSH descriptor: [Stress, Psychological] explode all trees 8877  #24 MeSH descriptor: [Outcome Assessment, Health Care] explode all trees 212663  #25 MeSH descriptor: [Patient Outcome Assessment] explode all trees 2408  #26 MeSH descriptor: [Treatment Outcome] explode all trees 201115  #27 MeSH descriptor: [Physician-Patient Relations] explode all trees 1915  #28 MeSH descriptor: [Health Personnel] explode all trees 15455  #29 MeSH descriptor: [Physicians] explode all trees 3883  #30 MeSH descriptor: [Nurses] explode all trees 1751  #31 MeSH descriptor: [Delivery of Health Care] explode all trees 67283  #32 MeSH descriptor: [Intensive Care Units, Pediatric] explode all trees 1718  #33 (quality NEXT of NEXT life) OR (life NEXT quality) OR hrqol OR wellbeing OR (Well NEXT Being) OR (ill NEXT being) OR satisfaction OR satisfied OR (Job NEXT Satisfaction) OR (work NEXT satisfaction) OR pain OR suffer* OR stress* OR distress* OR outcome* OR (Physician NEXT Patient NEXT Relation*) OR (Doctor NEXT Patient NEXT Relation*) OR (health NEXT personnel) OR (medical NEXT personnel) OR (Health NEXT Care NEXT Provider*) OR (Healthcare NEXT Provider*) OR (medical NEXT staff) OR (health NEXT care NEXT staff) OR (healthcare NEXT staff) OR (Health NEXT Care NEXT Professional*) OR (Healthcare NEXT Professional*) OR (medical NEXT professional*) OR physician* OR doctor* OR nurse* OR (Delivery NEXT of NEXT Health NEXT Care) OR (Delivery NEXT of NEXT Healthcare) OR (Health NEXT Care NEXT Delivery) OR (Healthcare NEXT Delivery) OR (Health NEXT Care NEXT System*) OR (Healthcare NEXT System*) OR (Pediatric NEXT Intensive NEXT Care NEXT Unit*) OR PICU OR (cardiac NEXT intensive NEXT care NEXT unit*) OR CICU 1115616  #34 #17 OR #18 OR #19 OR #20 OR #21 or #22 OR #23 OR #24 OR #25 OR #26 OR #27 OR #28 OR #29 OR #30 OR #31 OR #32 OR #33 1140945  #35 MeSH descriptor: [Heart Defects, Congenital] explode all trees 3195  #36 MeSH descriptor: [Aortic Coarctation] explode all trees 68  #37 MeSH descriptor: [Aortico-Ventricular Tunnel] explode all trees 0  #38 MeSH descriptor: [Arrhythmogenic Right Ventricular Dysplasia] explode all trees 15  #39 MeSH descriptor: [Bicuspid Aortic Valve Disease] explode all trees 20  #40 MeSH descriptor: [Cor Triatriatum] explode all trees 0  #41 MeSH descriptor: [Coronary Vessel Anomalies] explode all trees 22  #42 MeSH descriptor: [Anomalous Left Coronary Artery] explode all trees 0  #43 MeSH descriptor: [Bland White Garland Syndrome] explode all trees 0  #44 MeSH descriptor: [Myocardial Bridging] explode all trees 3  #45 MeSH descriptor: [Crisscross Heart] explode all trees 0  #46 MeSH descriptor: [Dextrocardia] explode all trees 17  #47 MeSH descriptor: [Ductus Arteriosus, Patent] explode all trees 392  #48 MeSH descriptor: [Ebstein Anomaly] explode all trees 3  #49 MeSH descriptor: [Ectopia Cordis] explode all trees 0  #50 MeSH descriptor: [Eisenmenger Complex] explode all trees 30  #51 MeSH descriptor: [Heart Septal Defects] explode all trees 455  #52 MeSH descriptor: [Truncus Arteriosus, Persistent] explode all trees 4  #53 MeSH descriptor: [Endocardial Cushion Defects] explode all trees 6  #54 MeSH descriptor: [Heart Septal Defects, Atrial] explode all trees 297  #55 MeSH descriptor: [Foramen Ovale, Patent] explode all trees 143  #56 MeSH descriptor: [Lutembacher Syndrome] explode all trees 0  #57 MeSH descriptor: [Double Outlet Right Ventricle] explode all trees 7  #58 MeSH descriptor: [Heterotaxy Syndrome] explode all trees 1  #59 MeSH descriptor: [Hypoplastic Left Heart Syndrome] explode all trees 97  #60 MeSH descriptor: [Isolated Noncompaction of the Ventricular Myocardium] explode all trees 0  #61 MeSH descriptor: [LEOPARD Syndrome] explode all trees 2  #62 MeSH descriptor: [Levocardia] explode all trees 0  #63 MeSH descriptor: [Quadricuspid Aortic Valve] explode all trees 0  #64 MeSH descriptor: [Tetralogy of Fallot] explode all trees 139  #65 MeSH descriptor: [Transposition of Great Vessels] explode all trees 84  #66 MeSH descriptor: [Congenitally Corrected Transposition of the Great Arteries] explode all trees 3  #67 MeSH descriptor: [Tricuspid Atresia] explode all trees 7  #68 MeSH descriptor: [Trilogy of Fallot] explode all trees 0  #69 MeSH descriptor: [Univentricular Heart] explode all trees 20  #70 MeSH descriptor: [Atrioventricular Block] explode all trees 134  #71 MeSH descriptor: [Cardiomyopathies] explode all trees and with qualifier(s): [congenital - CN] 2  #72 MeSH descriptor: [Pulmonary Arterial Hypertension] explode all trees 176  #73 MeSH descriptor: [Cardiology] explode all trees 294  #74 MeSH descriptor: [Heart Diseases] explode all trees 74093  #75 MeSH descriptor: [Arrhythmias, Cardiac] explode all trees 13630  #76 MeSH descriptor: [Carcinoid Heart Disease] explode all trees 2  #77 MeSH descriptor: [Cardiac Conduction System Disease] explode all trees 3804  #78 MeSH descriptor: [Cardiac Output, High] explode all trees 12  #79 MeSH descriptor: [Cardiac Output, Low] explode all trees 456  #80 MeSH descriptor: [Cardiac Tamponade] explode all trees 33  #81 MeSH descriptor: [Cardiomegaly] explode all trees 1992  #82 MeSH descriptor: [Cardiomyopathies] explode all trees 2696  #83 MeSH descriptor: [Cardiotoxicity] explode all trees 205  #84 MeSH descriptor: [Endocarditis] explode all trees 225  #85 MeSH descriptor: [Heart Valve Diseases] explode all trees 3206  #86 MeSH descriptor: [Pulmonary Atresia] explode all trees 5  #87 MeSH descriptor: [Myocardial Ischemia] explode all trees 38693  #88 MeSH descriptor: [Myocardial Stunning] explode all trees 83  #89 MeSH descriptor: [Pericardial Effusion] explode all trees 127  #90 MeSH descriptor: [Pericarditis] explode all trees 90  #91 MeSH descriptor: [Pneumopericardium] explode all trees 0  #92 MeSH descriptor: [Post-Cardiac Arrest Syndrome] explode all trees 14  #93 MeSH descriptor: [Postpericardiotomy Syndrome] explode all trees 19  #94 MeSH descriptor: [Pulmonary Heart Disease] explode all trees 80  #95 MeSH descriptor: [Rheumatic Heart Disease] explode all trees 180  #96 MeSH descriptor: [Ventricular Dysfunction] explode all trees 2913  #97 MeSH descriptor: [Ventricular Outflow Obstruction] explode all trees 1250  #98 MeSH descriptor: [Arrhythmia, Sinus] explode all trees 287  #99 MeSH descriptor: [Sick Sinus Syndrome] explode all trees 192  #100 MeSH descriptor: [Sinus Arrest, Cardiac] explode all trees 1  #101 MeSH descriptor: [Atrial Fibrillation] explode all trees 7226  #102 MeSH descriptor: [Atrial Flutter] explode all trees 482  #103 MeSH descriptor: [Bradycardia] explode all trees 693  #104 MeSH descriptor: [Brugada Syndrome] explode all trees 32  #105 MeSH descriptor: [Cardiac Complexes, Premature] explode all trees 468  #106 MeSH descriptor: [Commotio Cordis] explode all trees 0  #107 MeSH descriptor: [Heart Block] explode all trees 773  #108 MeSH descriptor: [Long QT Syndrome] explode all trees 435  #109 MeSH descriptor: [Pre-Excitation Syndromes] explode all trees 49  #110 MeSH descriptor: [Tachycardia] explode all trees 2267  #111 MeSH descriptor: [Ventricular Fibrillation] explode all trees 691  #112 MeSH descriptor: [Ventricular Flutter] explode all trees 2  #113 MeSH descriptor: [Cardiomyopathy, Dilated] explode all trees 670  #114 MeSH descriptor: [Hypertrophy, Left Ventricular] explode all trees 1128  #115 MeSH descriptor: [Hypertrophy, Right Ventricular] explode all trees 14  #116 MeSH descriptor: [Heart Failure] explode all trees 14455  #117 MeSH descriptor: [Cardio-Renal Syndrome] explode all trees 72  #118 MeSH descriptor: [Coronary Disease] explode all trees 18961  #119 MeSH descriptor: [Coronary Aneurysm] explode all trees 47  #120 MeSH descriptor: [Coronary Artery Disease] explode all trees 9329  #121 MeSH descriptor: [Coronary Occlusion] explode all trees 220  #122 MeSH descriptor: [Coronary Stenosis] explode all trees 2095  #123 MeSH descriptor: [Coronary Restenosis] explode all trees 1266  #124 MeSH descriptor: [Coronary-Subclavian Steal Syndrome] explode all trees 0  #125 MeSH descriptor: [Coronary Thrombosis] explode all trees 668  #126 MeSH descriptor: [Coronary Vasospasm] explode all trees 166  #127 MeSH descriptor: [Myocardial Infarction] explode all trees 15624  #128 MeSH descriptor: [Myocardial Reperfusion Injury] explode all trees 628  #129 (congenital NEXT heart NEXT disease*) OR CHD OR (congenital NEXT heart NEXT defect*) OR (congenital NEXT heart NEXT malformation*) OR (congenital NEXT heart NEXT abnormalit*) OR (congenital NEXT cardiac NEXT disease*) OR (congenital NEXT cardiac NEXT defect*) OR (congenital NEXT cardiac NEXT malformation*) OR (congenital NEXT cardiac NEXT abnormalit*) OR (congenital NEXT cardiovascular NEXT anomal*) OR (congenital NEXT cardiovascular NEXT malformation*) OR (congenital NEXT cardiovascular NEXT defect*) OR (Aortic NEXT Coarctation*) OR (Coarctation NEXT of NEXT the NEXT Aorta) OR (Coarctation NEXT of NEXT Aorta) OR (Aorta NEXT Coarctation*) OR (Aorta NEXT Dominant NEXT Coarctation) OR (Aortico-Ventricular NEXT Tunnel*) OR (Aortic NEXT Right NEXT Ventricular NEXT Tunnel*) OR (Aortic NEXT Left NEXT Ventricular NEXT Tunnel*) OR (Aortoventricular NEXT Tunnel*) OR (Aorto NEXT Ventricular NEXT Tunnel*) OR (Aorticoventricular NEXT Tunnel*) OR (Aortic NEXT Ventricular NEXT Tunnel*) OR (Aortico NEXT Left NEXT Ventricular NEXT Tunnel*) OR (Aorto NEXT Left NEXT Ventricular NEXT Tunnel*) OR (Aortico NEXT Right NEXT Ventricular NEXT Tunnel*) OR (Aorto NEXT Right NEXT Ventricular NEXT Tunnel*) OR (Arrhythmogenic NEXT Right NEXT Ventricular NEXT Dysplasia) OR (Arrhythmogenic NEXT Right NEXT Ventricular NEXT Cardiomyopathy NEXT Dysplasia) OR (ARVD-C) OR (Arrhythmogenic NEXT Right NEXT Ventricular NEXT Cardiomyopathy) OR (Arrhythmogenic NEXT Right NEXT Ventricular NEXT Dysplasia-Cardiomyopathy) OR (Bicuspid NEXT Aortic NEXT Valve NEXT Disease*) OR (Cor NEXT Triatriatum) OR (Subdivided NEXT Left NEXT Atrium*) OR (Triatrial NEXT Heart*) OR (Coronary NEXT Vessel NEXT Anomal*) OR (Anomalous NEXT Left NEXT Coronary NEXT Artery) OR (Bland NEXT White NEXT Garland NEXT Syndrome) OR ALCAPA OR (Myocardial NEXT Bridging*) OR (Myocardial NEXT Bridgings) OR (Crisscross NEXT Heart*) OR (Criss NEXT cross NEXT Heart*) OR Dextrocardia* OR (Patent NEXT Ductus NEXT Arteriosus) OR (Patency NEXT of NEXT the NEXT Ductus NEXT Arteriosus) OR (Ebstein NEXT Anomaly) OR (Ebstein's NEXT Malformation) OR (Ebstein NEXT Malformation) OR (Ebsteins NEXT Malformation) OR (Ebstein's NEXT Anomaly) OR (Ebsteins NEXT Anomaly) OR (Ectopia NEXT Cordis) OR (Eisenmenger NEXT Complex) OR (Eisenmenger's NEXT Complex) OR (Eisenmengers NEXT Complex) OR (Eisenmenger's NEXT Syndrome) OR (Eisenmengers NEXT Syndrome) OR (Eisenmenger NEXT Syndrome) OR (septal NEXT defect*) OR (Persistent NEXT Truncus NEXT Arteriosus) OR (Endocardial NEXT Cushion NEXT Defect*) OR (Persistent NEXT Common NEXT Atrioventricular NEXT Canal) OR (Persistent NEXT Ostium NEXT Primum) OR (Patent NEXT Oval NEXT Foramen) OR (Patent NEXT Foramen NEXT Ovale) OR (Lutembacher NEXT Syndrome) OR (Lutembacher's NEXT Syndrome) OR (Lutembachers NEXT Syndrome) OR (Double NEXT Outlet NEXT Right NEXT Ventricle*) OR (Taussig NEXT Bing NEXT Anomaly) OR (Heterotaxy NEXT Syndrome*) OR (Visceral NEXT Heterotax*) OR (Situs NEXT Ambiguus) OR (Polysplenia NEXT Syndrome*) OR (Left NEXT Atrial NEXT Isomerism NEXT with NEXT Polysplenia) OR (Right NEXT Atrial NEXT Isomerism*) OR (Asplenia NEXT Syndrome*) OR (Right NEXT Atrial NEXT Isomerism NEXT with NEXT Asplenia) OR (Asplenia NEXT with NEXT Cardiovascular NEXT Anomalies) OR (Ivemark NEXT Syndrome) OR (Left NEXT Atrial NEXT Isomerism*) OR (Hypoplastic NEXT Left NEXT Heart NEXT Syndrome) OR (Left NEXT Heart NEXT Hypoplasia NEXT Syndrome) OR (Isolated NEXT Noncompaction NEXT of NEXT the NEXT Ventricular NEXT Myocardium) OR (Isolated NEXT Non-compaction NEXT of NEXT the NEXT Ventricular NEXT Myocardium) OR (Noncompaction NEXT of NEXT the NEXT Left NEXT Ventricular NEXT Myocardium) OR (LEOPARD NEXT Syndrome) OR (Lentiginosis NEXT Cardiomyopathic) OR (Progressive NEXT Cardiomyopathic NEXT Lentiginos*) OR (Cardio NEXT Cutaneous NEXT Syndrome) OR (Cardiomyopathic NEXT Lentiginos*) OR Levocardia OR (Quadricuspid NEXT Aortic NEXT Valve*) OR (Quadricuspid NEXT Aortic NEXT Valvular NEXT Disease) OR (Tetralogy NEXT of NEXT Fallot) OR (Fallot NEXT Tetralogy) OR (Fallot's NEXT Tetralogy) OR (Fallots NEXT Tetralogy) OR (Transposition NEXT of NEXT Great NEXT Vessels) OR (Great NEXT Vessels NEXT Transposition*) OR (Transposition NEXT of NEXT the NEXT Great NEXT Arteries) OR (Great NEXT Arteries NEXT Transposition*) OR (Congenitally NEXT Corrected NEXT Transposition*) OR (Tricuspid NEXT Atresia*) OR (Tricuspid NEXT Valve NEXT Atresia*) OR (Trilogy NEXT of NEXT Fallot) OR (Fallot NEXT Trilogy) OR (Fallot's NEXT Trilogy) OR (Fallots NEXT Trilogy) OR (Univentricular NEXT Heart*) OR (Complex NEXT Single NEXT Ventricle*) OR (congenital NEXT heart NEXT block) OR (Atrioventricular NEXT Block) OR (congenital NEXT cardiac NEXT block) OR (congenital NEXT cardial NEXT block) OR (Supravalvar NEXT Aortic NEXT Stenosis NEXT Syndrome) OR (Hypercalcemia NEXT Supravalvar NEXT Aortic NEXT Stenosis) OR (Pulmonary NEXT Arterial NEXT Hypertension) OR (Pulmonary NEXT Artery NEXT Hypertension) OR cardiology OR (Heart NEXT Disease*) OR (heart NEXT disorder) OR (cardiac NEXT disease*) OR (cardiac NEXT disorder*) OR arrhythmia* OR dysrhythmia* OR (Carcinoid NEXT Heart NEXT Disease*) OR (cardiac NEXT conduction) OR (high NEXT cardiac NEXT output) OR (low NEXT cardiac NEXT output) OR (Cardiac NEXT Tamponade*) OR (Pericardial NEXT Tamponade*) OR Cardiomegaly OR (Enlarged NEXT Heart*) OR (Cardiac NEXT Hypertroph*) OR (Heart NEXT Hypertroph*) OR Cardiomyopath* OR Myocardiopath* OR (Myocardial NEXT Disease*) OR Cardiotoxicit* OR (Cardiac NEXT Toxicit*) OR Endocarditis OR (Heart NEXT Valve NEXT Disease*) OR (valve NEXT disease*) OR (valve NEXT insufficiency) OR (valve NEXT stenosis) OR (valve NEXT prolapse) OR (valve NEXT atresia) OR (pulmonary NEXT atresia) OR (Myocardial NEXT Ischemia*) OR (Ischemic NEXT Heart NEXT Disease*) OR (Myocardial NEXT Stunning) OR (Myocardial NEXT Hibernation) OR (Stunned NEXT Myocardium) OR (Pericardial NEXT Effusion*) OR Hemopericardium OR Chylopericardium OR Pericarditis OR Pleuropericarditis OR Pneumopericardium OR (Post-Cardiac NEXT Arrest NEXT Syndrome*) OR (Postresuscitation NEXT Disease*) OR (Postcardiac NEXT Arrest NEXT Syndrome*) OR (Postpericardiotomy NEXT Syndrome*) OR (Postcommissurotomy NEXT Syndrome*) OR (Pulmonary NEXT Heart NEXT Disease*) OR (Rheumatic NEXT Heart NEXT Disease*) OR (Bouillaud NEXT Disease) OR (Bouillaud's NEXT Disease) OR (Bouillauds NEXT Disease) OR (Ventricular NEXT Dysfunction*) OR (Ventricular NEXT Outflow NEXT Obstruction) OR (Sinus NEXT Arrhythmia*) OR (Sinoatrial NEXT Arrhythmia*) OR (Sick NEXT Sinus NEXT Syndrome) OR (Sick NEXT Sinus NEXT Node NEXT Syndrome) OR (Sinus NEXT Node NEXT Dysfunction) OR (Sinus NEXT Node NEXT Disease*) OR (Cardiac NEXT Sinus NEXT Arrest*) OR (Cardiac NEXT Sinus NEXT Pause*) OR (Atrial NEXT Fibrillation*) OR (Auricular NEXT Fibrillation*) OR (Atrial NEXT Flutter*) OR (Auricular NEXT Flutter*) OR Bradycardia* OR Bradyarrhythmia* OR (Brugada NEXT Syndrome) OR (Sudden NEXT Unexplained NEXT Death NEXT Syndrome) OR (Sudden NEXT Unexplained NEXT Nocturnal NEXT Death NEXT Syndrome) OR (Brugada NEXT ECG NEXT Pattern) OR (Brugada NEXT Type NEXT ECG NEXT Pattern) OR (Premature NEXT Beats*) OR Extrasystole OR (Premature NEXT Cardiac NEXT Complex) OR (Ectopic NEXT Heartbeat*) OR (Commotio NEXT Cordis) OR (Cardiac NEXT Concussion*) OR (Heart NEXT Block*) OR (Auriculo-Ventricular NEXT Dissociation*) OR (Atrioventricular NEXT Dissociation*) OR (A NEXT V NEXT Dissociation*) OR (Long NEXT QT NEXT Syndrome) OR Parasystole* OR (Pre-Excitation NEXT Syndrome*) OR (Preexcitation NEXT Syndrome*) OR Tachycardia* OR Tachyarrhythmia* OR (Ventricular NEXT Fibrillation*) OR (Ventricular NEXT Flutter*) OR (Dilated NEXT Cardiomyopath*) OR (Idiopathic NEXT Cardiomyopath*) OR (Congestive NEXT Cardiomyopath*) OR (ventricular NEXT hypertroph*) OR (Hypertrophic NEXT Cardiomyopath*) OR (Heart NEXT Failure) OR (Cardio-Renal NEXT Syndrome*) OR (Reno-Cardiac NEXT Syndrome*) OR (Renocardiac NEXT Syndrome*) OR (Cardiorenal NEXT Syndrome*) OR (Coronary NEXT Disease*) OR (Coronary NEXT Aneurysm*) OR (Coronary NEXT Artery NEXT Disease*) OR (Left NEXT Main NEXT Disease*) OR (Coronary NEXT Arterioscleros*) OR (Coronary NEXT Atheroscleros*) OR (Coronary NEXT Occlusion*) OR (Coronary NEXT Stenos*) OR (Coronary NEXT Artery NEXT Stenos*) OR (Coronary NEXT Restenos*) OR (Coronary-Subclavian NEXT Steal NEXT Syndrome*) OR (Coronary NEXT Thrombos*) OR (Coronary NEXT Vasospasm*) OR (Coronary NEXT Artery NEXT Spasm*) OR (Coronary NEXT Spasm*) OR (Myocardial NEXT Infarct**) OR (Cardiovascular NEXT Stroke*) OR (Heart NEXT Attack*) OR (Myocardial NEXT Reperfusion NEXT Injur*) OR (Myocardial NEXT Ischemic NEXT Reperfusion NEXT Injur*) 164688  #130 #35 OR #36 OR #37 OR #38 OR #39 OR #40 OR #41 OR #42 OR #43 OR #44 OR #45 OR #46 OR #47 OR #48 OR #49 OR #50 OR #51 OR #52 OR #53 OR #54 OR #55 OR #56 OR #57 OR #58 OR #59 OR #60 OR #61 OR #62 OR #63 OR #64 OR #65 OR #66 OR #67 OR #68 OR #69 OR #70 OR #71 OR #72 OR #73 OR #74 OR #75 OR #76 OR #77 OR #78 OR #79 OR #80 OR #81 OR #82 OR #83 OR #84 OR #85 OR #86 OR #87 OR #88 OR #89 OR #90 OR #91 OR #92 OR #93 OR #94 OR #95 OR #96 OR #97 OR #98 OR #99 OR #100 OR #101 OR #102 OR #103 OR #104 OR #105 OR #106 OR #107 OR #108 OR #109 OR #110 OR #111 OR #112 OR #113 OR #114 OR #115 OR #116 OR #117 OR #118 OR #119 OR #120 OR #121 OR #122 OR #123 OR #124 OR #125 OR #126 OR #127 OR #128 OR #129 169197  #131 #8 AND #16 AND #34 AND #130 454  Limit to trials: 181 |
